# Association of malalignment, muscular dysfunction, proprioception, laxity and abnormal joint loading with tibiofemoral knee osteoarthritis - a systematic review and meta-analysis

**DOI:** 10.1186/s12891-018-2202-8

**Published:** 2018-07-28

**Authors:** Joyce A. C. van Tunen, Andrea Dell’Isola, Carsten Juhl, Joost Dekker, Martijn Steultjens, Jonas B. Thorlund, Hans Lund

**Affiliations:** 1 Research Unit for Musculoskeletal Function and Physiotherapy, Department of Sports Science and Clinical Biomechanics, Faculty of Health Sciences, University of Southern Denmark, Campusvej 55, 5230 Odense, Denmark; 2 School of Health and Life Sciences, Glasgow Caledonian University, Glasgow, Scotland; 3 Department of Rehabilitation, Copenhagen University Hospital, Herlev and Gentofte, Denmark; 4 Department of Rehabilitation Medicine and Department of Psychiatry, EMGO Insitute for Health and Care Research, VU University Medical Center, Amsterdam, the Netherlands; 5 Centre for Evidence Based Practice, Western Norway University of Applied Sciences, Bergen, Norway

**Keywords:** Osteoarthritis, Knee, Biomechanics, Systematic review, Meta-analysis

## Abstract

**Background:**

To investigate (1) the association of specific biomechanical factors with knee osteoarthritis and knee osteoarthritis development, and (2) the impact of other relevant risk factors on this association.

**Methods:**

MEDLINE, EMBASE, CINAHL and SPORTDiscus were searched up until April 2017. Studies were included if they fulfilled the following criteria: the study 1) assessed the association of a biomechanical factor with knee osteoarthritis, or knee osteoarthritis development; 2) reported on skeletal malalignment, muscular dysfunction, impaired proprioception, laxity and abnormal loading during gait; 3) was a cohort study with participants developing knee osteoarthritis and participants not developing knee osteoarthritis, or a case-control or cross-sectional study with participants with knee osteoarthritis and without knee osteoarthritis. Risk of bias was assessed with the QUIPS tool and meta-analyses were performed using random effects models.

**Results:**

Of 6413 unique studies identified, 59 cross-sectional studies were eligible for meta-analyses (9825 participants, 5328 with knee osteoarthritis). No cohort studies fulfilled the inclusion criteria. Compared with healthy controls, patients with knee osteoarthritis have higher odds of having lower muscle strength, proprioception deficits, more medial varus-valgus laxity and less lateral varus-valgus laxity. Patients with medial knee osteoarthritis have higher odds of having a higher knee adduction moment than healthy controls. Level of evidence was graded as ‘very low’ to ‘moderate’ quality. Due to large between study differences moderation of other risk factors on biomechanical risk factors could not be evaluated.

**Conclusions:**

Patients with knee osteoarthritis are more likely to display a number of biomechanical characteristics. The causal relationship between specific biomechanical factors and the development of knee osteoarthritis could not be determined as no longitudinal studies were included. There is an urgent need for high quality, longitudinal studies to evaluate the impact of specific biomechanical factors on the development of knee osteoarthritis.

**Trial Registration:**

(PROSPERO ID: CRD42015025092).

**Electronic supplementary material:**

The online version of this article (10.1186/s12891-018-2202-8) contains supplementary material, which is available to authorized users.

## Background

Tibiofemoral knee osteoarthritis (OA) is mainly considered a mechanically driven disease [1] and numerous interventions such as braces, insoles and exercise therapy aim at modifying potential biomechanical drivers to prevent development or stall progression [2]. Biomechanical knee joint-related factors that often are subject to research in relation to knee OA are skeletal malalignment, muscular dysfunction, impaired proprioception, laxity and abnormal loading during gait [3]. The association of those biomechanical factors and other relevant risk factors (e.g. age, gender, obesity, knee injury) with knee OA and its onset have been reported in many individual studies. Such studies describing biomechanical factors’ association with the presence or development of knee OA are often used to justify specific research questions but may not necessarily be representative of the available literature. Systematic reviews and meta-analyses give an overview the complete evidence of relevant biomechanical factors and their association with knee osteoarthritis.

Some previous reviews have attempted to summarize the evidence for the association between malalignment, muscular dysfunction, impaired proprioception, and laxity with knee OA. However, several of these reviews are not up-to-date and no attempts were made to estimate the magnitude of association of these biomechanical factors with knee OA [4–8]. Whereas systematic reviews including meta-analyses on knee extensor strength and knee joint loading (i.e. knee adduction moment) have primarily focussed on biomechanical risk factors for onset and progression of knee OA [9–11], only one study has systematically compared gait biomechanics in knee OA patients with controls quantitatively [12]. Thus, it has recently been emphasized that systematic reviews and meta-analysis to investigate the relationship between different biomechanical risk factors and OA should be performed [13].

In this systematic review and meta-analysis we aimed to (1) investigate the association of skeletal malalignment, muscular dysfunction, impaired proprioception, laxity and abnormal loading during gait with knee OA and knee OA development, and (2) to investigate the impact of other relevant risk factors on this association. Such knowledge is important when framing future research questions and designing targeted biomechanical interventions.

## Methods

### Registration and ethics

The protocol for this systematic review and meta-analysis [14] has been published previously and has been registered with the International Prospective Register of Systematic Reviews (PROSPERO ID: CRD42015025092). This systematic review and meta-analysis follows the Cochrane Collaboration guideline for preparing systematic review and meta-analysis and are reported according to the Preferred Reporting Items for Systematic Reviews and Meta-analyses (PRISMA) guideline [15].

### Definition of biomechanical domains

We defined skeletal malalignment as abnormal alignment between the femur and the tibia in the frontal plane (i.e. varus or valgus alignment) [8]. Muscle dysfunction indicates muscle weakness, loss of muscle endurance or changed muscle activation patterns for the muscles that act on the knee joint [3, 5, 6]. Impaired proprioception refers to deterioration of the ability to detect knee joint position and movement [4]. Laxity is a loss of passive joint stabilisation due to the inability of passive structures in and around the knee (knee ligaments, cruciate ligaments, capsule) to provide an adequate counterbalance to the mechanical forces acting upon the knee during activity [7]. Abnormal loading during gait is often represented by evaluating external knee joint moments or the occurrence of varus or valgus thrust [9].

### Search strategy

MEDLINE, EMBASE, SPORTDiscus, and the Cumulative Index to Nursing and Allied Health Literature Database (CINAHL) were searched from their inception until April 2017. Searches used subject headings (MeSH) and text words related to osteoarthritis, biomechanical factors and study types. The complete search strategy can be found in the study protocol [14].

### Study selection

Studies were included if they fulfilled the following criteria: 1) the study assessed the association of a biomechanical factor with knee OA or incident knee OA development; 2) the biomechanical factor in the study was a knee joint related factor that interacts with the forces, moments and kinematics in and around a synovial joint (e.g. skeletal malalignment, proprioception, etc.); 3) the study was a cohort study with participants developing knee OA and participants not developing knee OA, or the study was a case-control or cross-sectional study with participants with knee OA and without knee OA. Studies were excluded if: 1) the study only included participants with patellofemoral osteoarthritis; 2) the study did not distinguish between hip osteoarthritis and knee OA; 3) study participants underwent treatment such as rehabilitation or surgery; 4) the study did not define knee OA in accordance to the criteria described in the protocol; 5) the study compared knees from the same participant (i.e. compared one knee with, and one knee without osteoarthritis within the same participant); 6) both knees are assessed in patients with bilateral knee OA. A detailed description of the eligibility criteria can be found in the study protocol [14].

Two reviewers (JT, ADI) independently screened eligibility of titles and abstracts of the studies obtained by the search. Subsequently, the reviewers used a standardized form to select studies eligible for inclusion in the review based on full text. Consensus was reached by discussion.

### Assessment of risk of bias

Risk of bias was scored independently by two reviewers (JT, ADI) using the Quality In Prognostic Studies (QUIPS) tool [16] as described in the protocol [14]. Six areas of potential study biases were assessed: study participation, study attrition, prognostic factor measurement, study confounding, outcome measurement, and statistical analysis and reporting. Attrition was not applicable for cross-sectional studies.

### Data extraction

Two reviewers (JT, ADI) used a customised, pilot-tested form to extract data from the included studies. The following information was extracted by both reviewers: authors, publication year, number of participants developing and not developing knee OA (in cohort studies) or number of participants in the knee OA and control group (in cross-sectional studies), sex, age, body mass index (BMI in kg/m^2^), knee injury, knee OA definition, radiographic disease severity, involved compartment and examined biomechanical factor.

For malalignment and thrust measurements, the number of patients and controls with and without malalignment/thrust were extracted. If only the group mean and standard deviation was reported for malalignment, we transformed this into the number of participants with varus (or valgus) alignment and the number of participants with neutral and valgus (or varus) alignment by use of the normal deviation, where more than 1 degree deviation signifies abnormal alignment. For muscular dysfunction, impaired proprioception, laxity and external knee joint moments, group mean and standard deviation were extracted for patients and controls. For all factors, odds ratios were extracted if this was the only available data.

Data was grouped per study design, and into the five biomechanical domains. To allow for comparison, data for each biomechanical factor was further divided. Skeletal malalignment was subdivided into varus and valgus alignment, and muscle dysfunction was subdivided into extensor and flexor weakness. Impaired proprioception was subdivided into reposition error, and thresholds to detect a passive movement in the sagittal or frontal plane (varus and valgus direction). Laxity was subdivided into varus-valgus laxity measured at the medial and lateral side, and anterior-posterior laxity. Abnormal loading was subdivided into varus thrust, valgus thrust, knee adduction moment (KADM), knee flexion moment (KFM), etc.. One outcome measure per study for knee extensor and flexor strength was selected based on hierarchies described by Hall et al. [17] and Øiestadt et al. [10]. Measurements of abnormal loading during gait had to be reported as the peak or maximal value, or had to be examined during the same time period of the gait cycle. Varus and valgus thrust were assessed during early stance. KADM was assessed during early stance or midstance. KFM was assessed during midstance, while knee extension moment (KEM) was assessed during terminal stance.

### Statistical analysis

Mean and standard deviation on continuous scales were transformed to odds ratio (OR) using the Chinn formula as described in the Cochrane Handbook [18]. Meta-analyses using random effects models were applied on the (logarithmic transformed) OR of developing knee OA in participants with the biomechanical factor of interest (cohort studies), or the (logarithmic transformed) OR of the biomechanical factor being present in participants with knee OA compared to the control group (cross-sectional studies). Meta-regression analyses were used to assess the impact of other risk factors (i.e. age, gender, BMI, knee injury) and radiographic severity on the association of biomechanical risk factors with knee OA or knee OA development.

Heterogeneity between studies was examined with standard Q-tests, and calculated as the I^2^ statistics. Secondary analyses were described in the protocol [14]. The Grading of Recommendation, Assessment, Development and Evaluation (GRADE) framework was used to evaluate quality of evidence [19].

## Results

**Fig. 1:**
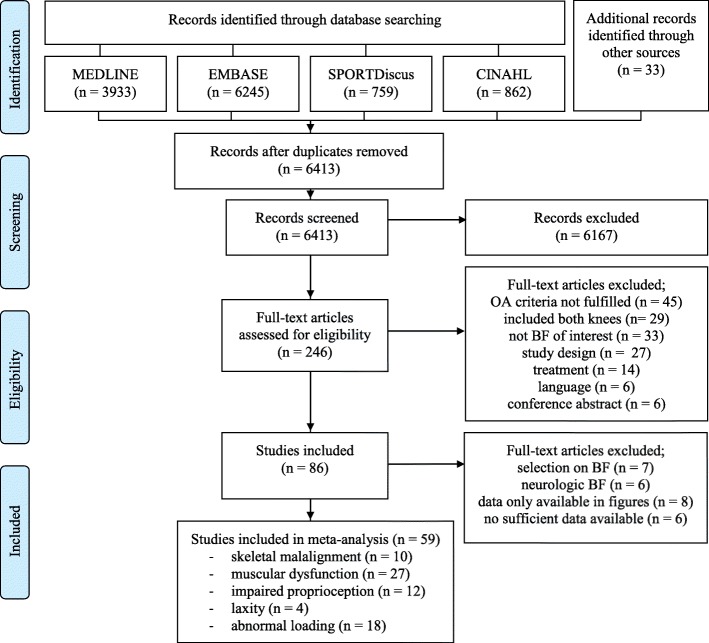
Flow diagram of study selection. Other sources were reference lists of included papers and suggestions from experts in the field. OA: osteoarthritis, BF: biomechanical factor

A total of 6413 unique studies were identified. Eighty-six studies met all eligibility criteria and 59 were included in meta-analyses, as 27 studies did not provide sufficient data for the meta-analysis (Fig. 1).

Only cross-sectional studies were included, as no longitudinal studies fulfilled the in- and exclusion criteria. Therefore, we could not report on the association of specific biomechanical factors and knee OA development. Due to sparse data for most meta-analyses it was not feasible to investigate the impact (moderation) of other relevant risk factors on any associations, thus no meta-regression analyses were performed. Few studies reported data on biomechanical outcomes for different level of radiographic severity, precluding any sub group analysis based on disease severity [20–23]. Therefore, we present a comprehensive overview of the direction and magnitude of associations between knee OA and biomechanical factors. Duplicate data extraction on 86 studies was deemed not reasonable time wise, thus we performed duplicate data extraction on half of the studies (JT/ADI), and single data extraction on the other half (JT).

### Study characteristics

The 59 cross-sectional studies for meta-analyses included a total of 9825 participants (5328 patients with knee OA and 4497 healthy controls). 57% of the knee OA patients and controls were women. Mean age was 63.5 and 61.4 years for knee OA patients and controls, respectively. Mean BMI (kg/m^2^) was 29.1 for knee OA patients and 26.5 for controls. As few studies included information on previous knee injury, and knee injury was often used as an exclusion criterion for knee OA patients, healthy controls, or both, we have not included information on this. Sixteen studies reported radiographic severity. Twenty-one studies assessed patients with medial knee OA, 37 with a combination of both compartments or the involved compartment was not specified, and one study assessed patients with medial and lateral knee OA separately.

Skeletal malalignment was assessed in 10 studies (*n* = 1051) [24–33], muscular dysfunction in 27 studies (*n* = 6086) [20, 21, 23–25, 27, 34–54], impaired proprioception in 12 studies (*n* = 565) [34, 37, 46, 54–62], laxity in four studies (*n* = 321) [22, 23, 27, 63], and abnormal loading in 18 studies (*n* = 5974) [25, 27, 50, 64–78] (Table 1). A summary of findings table can be found in Additional file 1, and forest plots of data pooling for each of the domains and the presence of knee OA can be found in Additional file 2.

**Table 1:** Study characteristics

Study	No. OA and control (% female)	Group age, mean ± SD	Group BMI, mean ± SD	Definition OA	Radiographic severity OA	Compartment	Biomechanical factor	Risk of bias
Nordesjö (1983)	OA:14 (85.7) C:96 (47.9)	OA:68 C: NR	NR	Radiographic	NR	Medial / lateral OA	MUSC (extensor weakness; flexor weakness)	High
Hall (1993)	OA:21 (81.0) C:21 (81.0)	OA:62.6 ± 13.9 C:62.2 ± 13.8	OA:29.6 C:25.5	Symptomatic	NR	Medial / lateral OA	MUSC (extensor weakness; flexor weakness)	High
Marks (1993)	OA:10 (100) C:10 (100)	OA: 54.6 ± 9.87 C:48.2 ± 6.97	OA:30.14 C:24.39	Symptomatic	NR	Medial / lateral OA	PROP (reposition error)	High
Marks (1996)	OA:15 (100) C:11 (100)	OA:60.07 ± 10.74 C:48.63 ± 6.77	NR	Symptomatic	NR	Medial / lateral OA	PROP (reposition error)	High
Cooke (1997)	OA:167 (54.4) C:40 (65)	OA:66 ± 12 C:66 ± 8	NR	Radiographic and symptomatic	NR	Medial / lateral OA	MAL (varus)	High
Pai (1997)	OA:30 (73.3) C:29 (58.6)	OA:68.2 ± 8.3 C:71.3 ± 8.3	OA:28.6 ± 4.1 C:24.9 ± 3.9	Radiographic	KL2: 10, KL3: 13, KL4: 7	Medial / lateral OA	PROP (TDPM)	Low
Sharma (1997)	OA:28 (53.6) C:29 (55.2)	OA:65.5 ± 14.5 C:71 ± 11.5	OA:28.9 ± 5.8 C:26.3 ± 5.7	Radiographic	KL2: 7, KL3: 12, KL4: 9	Medial / lateral OA	PROP (TDPM)	Low
Garsden (1999)	OA:20 (50) C:20 (50)	OA:62.8 (R 50–73) C:60.8 (R 51–71)	NR	Radiographic and symptomatic	NR	Medial / lateral OA	PROP (reposition error)	High
Cheing (2001)	OA:66 (86.4) C:10 (50)	OA: women 63.5 ± 7.8; men 63.9 ± 8.9 C: women 57.4 ± 6.6; men 62.4 ± 8.4	OA: women 27.7 ± 4.2; men 26.3 ± 3.5 C: women 24.4 ± 1.8; men 25.5 ± 4.8	Radiographic	NR	Medial / lateral OA	MUSC (extensor weakness; flexor weakness)	Low
Kaufman (2001)	OA:139 (66.2) C:20 (55)	OA:57 ± 12.5 C:30 ± 8	OA:30.48 C:25.06	Radiographic and symptomatic	NR	Medial / lateral OA	LOAD (KEM; KADM; KABM; KIRM; KERM)	Low
Hortobagyi (2004)	OA:20 (75) C:20 (75)	OA:57.5 ± 7.3 C:56.8 ± 5	OA:29.3 ± 3.2 C:28.3 ± 3	Radiographic	NR	Medial / lateral OA	PROP (reposition error)	Low
Lewek (2004)	OA:12 (41.7) C:12 (50.0)	OA:52.6 ± 7.2 C:48.9 ± 4.9	OA:31.3 ± 5.1 C:28.6 ± 5.6	Radiographic and symptomatic	NR	Medial OA	MUSC (extensor weakness)	High
Lewek (2005)	OA:21 (33.3) C:19 (36.8)	OA:49.3 ± 7 C:49.3 ± 5.8	OA:30 ± 4.3 C:28.3 ± 4.9	Radiographic and symptomatic	NR	Medial OA	Laxity (varus-valgus: medial; varus-valgus:lateral)	High
Aoda (2006)	OA:688 (NR) C:568 (NR)	NR	NR	Radiographic	KL2: 510, KL3: 136, KL4: 42	Medial / lateral OA	LOAD (varus thrust)	High
Hall (2006)	OA with pain:36 (61.1) OA without pain:23 (69.6) C: 55 (69.1)	OA with pain: 68.78 ± 7.8 OA without pain: 69.22 ± 5.78 C:67.49 ± 8.45	OA with pain: 29.72 ± 4.4 OA without pain: 28.45 ± 4.69 C:25.89 ± 3.97	Radiographic and symptomatic	NR	Medial / lateral OA	MUSC (extensor weakness); PROP (reposition error)	Low
Zhai (2006)	OA:239 (23.4) C:261 (17.6)	OA:62.7 ± 7.1 C:63.0 ± 7.4	OA:28.7 ± 5.3 C:26.9 ± 4.1	Symptomatic	NR	Medial / lateral OA	MUSC (extensor weakness)	High
Ling (2007)	OA:17 (NR) C:22 (NR)	NR	NR	Radiographic	KL2: 4, KL3: 9, KL4: 4	Medial / lateral OA	MUSC (extensor weakness)	Low
Petterson (2007)	OA:44 (56.8) C:44 (56.8)	OA:62.3 ± 6.8 C:61.3 ± 7.7	OA:27.9 ± 3.9 C:26.8 ± 4.4	Symptomatic	NR	Medial / lateral OA	MUSC (extensor weakness)	High
Rudolph (2007)	OA:15 (46.7) C:15 (46.7)	OA:49.2 (R 39–57) C:49.2 (R 40–57)	OA:30.7 ± 4.8 C:28.7 ± 5.5	Symptomatic	NR	Medial OA	MAL (varus)	High
Schmitt (2007)	OA:28 (50) C:26 (50)	OA: 60.4 (R 39–78) C: 58.5 (R 38–76)	OA:31.84 C:29.74	Radiographic	NR	Medial OA	MAL (varus); MUSC (extensor weakness); Laxity (varus-valgus: medial; varus-valgus:lateral); LOAD (KFM; KEM: KADM)	Low
Thorp (2007)	OA:52 (73.1) C:37 (73.0)	OA:56.2 ± 10.4 C:53.6 ± 6.1	OA:29.1 ± 3.9 C:26.7 ± 3.9	Radiographic	NR	Medial OA	LOAD (KADM)	Low
Zhai (2007)	OA:56 (NR) C:259 (NR)	NR	NR	Radiographic	NR	Medial / lateral OA	MAL (varus; valgus)	High
Astephen (2008)	OA:61 (54.1) C:60 (61.7)	OA:64.49 ± 7.75 C:50.27 ± 10.09	OA:32.05 ± 5.48 C:25.45 ± 4.04	Radiographic and symptomatic	NR	Medial / lateral OA	LOAD (KFM; KIRM)	High
Janakiramanan (2008)	OA:74 (64) C:128 (78)	OA:59.4 ± 8.5 C:63.8 ± 10	OA:29.4 ± 5.3 C:26.8 ± 4.8	Radiographic	NR	Medial / lateral OA	MAL (valgus)	Low
Liikavainio (2008)	OA:54 (0) C:53 (0)	OA:59 ± 5.3 C:59.2 ± 4.7	OA:29.7 ± 4.7 C:27.1 ± 3.1	Symptomatic	KL1: 12, KL2: 15, KL3: 19, KL4: 8^d^	Medial / lateral OA	MUSC (extensor weakness; flexor weakness)	Low
Lund (2008)	OA:21 (100) C:29 (100)	OA:57.1 ± 12 C:55.3 ± 10.1	OA:28.5 ± 7.4 C:23.3 ± 7.4	Radiographic and symptomatic	NR	Medial / lateral OA	PROP (reposition error; TDPM)	Low
Mohammadi (2008)	OA:30 (100) C:30 (100)	OA:46.4 ± 5.1 C:45.4 ± 4.9	OA:27.1 ± 3.3 C:26.9 ± 4.3	Symptomatic	KL1/2: 5, KL3/4: 26^d, e^	Medial / lateral OA	MUSC (extensor weakness); PROP (reposition error)	Low
Wu (2008)	OA:11 (72.7) C:10 (70.0)	OA:61.1 ± 10.3 C:61.4 ± 6.5	OA:24.4 ± 2.4 C:23.2 ± 2.2	Radiographic	NR	Medial / lateral OA	MUSC (extensor weakness)	High
Heiden (2009a)	OA:54 (55.6) C:27 (66.7)	OA:65.6 ± 7.6 C:64.2 ± 5.1	OA:28.1 ± 4.2 C:24.4 ± 3.6	Radiographic	NR	Medial / lateral OA	LOAD (KEM; KADM)	Low
Heiden (2009b)	OA:54 (55.6) C:27 (66.7)	OA:57 ± 10 C:56 ± 15	OA:29 ± 7 C:25 ± 4	Radiographic	NR	Medial / lateral OA	MUSC (extensor weakness)	Low
Chang (2010)	OA:2026 (57.5) C:1566 (58.7)	OA:63.4 ± 9.1 C:61.0 ± 8.9	OA:29.4 ± 4.8 C:27.6 ± 4.6	Radiographic	NR	Medial / lateral OA	MUSC (extensor weakness); LOAD (valgus and varus thrust)	Low
Cibere (2010)	OA:98 (53) C:33 (52)	OA M:65 C M:47	OA M:26.6 C M:24.1	Symptomatic	NR	Medial / lateral OA	MAL (varus; valgus)	High
Dixon (2010)	OA:37 (62) C:11 (64)	OA: 65.6 ± 9.2 C:57.3 ± 9.5	OA:29.5 ± 4.1 C:25.3 ± 3.1	Radiographic and symptomatic	KL1: 3, KL2: 9, KL3: 10, KL4: 15^d^	Medial OA	LOAD (KEM)	High
Foroughi (2010)	OA:17 (100) C:17 (100)	OA:66 ± 8 C:51 ± 7	OA:26 ± 3 C:25 ± 4	Symptomatic	NR	Medial OA	MAL (varus); MUSC (extensor weakness); LOAD (KADM)	Low
Linley (2010)	OA:40 (57.5) C:40 (57.5)	OA:63 ± 10 C:64 ± 9	OA:27.4 ± 5.5 C:24 ± 3.2	Radiographic and symptomatic	KL1: 4, KL2: 20, KL3: 9, KL4: 7^d^	Medial OA	LOAD (KADM)	Low
Creaby (2010)	mild OA: 50 (54.0) moderate OA: 45 (44.4) severe OA: 32 (34.4) C:32 (53.1)	mild OA: 61.61 ± 7.12 moderate OA: 65.23 ± 7.72 severe OA: 66.4 ± 9.39 C:59.39 ± 6.92	mild OA: 28.37 ± 4.72 moderate OA: 30.03 ± 4.31 severe OA: 29.72 ± 5.2 C:26.62 ± 2.82	Radiographic and symptomatic	NR	Medial OA	Laxity (varus-valgus: medial; varus-valgus:lateral)	Low
Berger (2011)	OA:8 (50) C:8 (50)	OA:61.3 ± 3.8 C:61.8 ± 5.9	OA:33.4 ± 4.6 C:27.0 ± 27.3^1^	Radiographic and symptomatic	NR	Medial / lateral OA	MUSC (extensor weakness)	High
Butler (2011)	MOA:15 (NR) LOA:15 (NR) C:15 (NR)	MOA:66.2 ± 7.8 LOA:65.7 ± 6.4 C:56.3 ± 10.7	MOA:32.2 ± 7.9 LOA:30.4 ± 7.5 C:27.8 ± 5.7	Radiographic	MOA: KL2: 5, KL3: 4, KL4: 6 LOA: KL2: 3, KL3: 5, KL4: 7	Medial and lateral OA	LOAD (KADM)	Low
Cammarata (2011)	OA:13 (46.2) C:14 (50)	OA:57 ± 10 C:56 ± 15	OA:29 ± 7 C:25 ± 4	Radiographic and symptomatic	NR	Medial / lateral OA	MUSC (extensor weakness; flexor weakness)	Low
Zeni (2011)	OA:30 (NR) C:15 (NR)	OA:63 ± 7 C:58 ± 9	OA:29.75 ± 4.2 C:25.58 ± 3.5	Radiographic	NR	Medial OA	MUSC (extensor weakness, flexor weakness)	High
Cammarata (2012)	OA:13 (46.2) C:14 (50)	OA:57 ± 10 C:56 ± 15	OA:29 ± 7 C:25 ± 4	Radiographic and symptomatic	NR	Medial / lateral OA	PROP (TDPM-varus; TDPM-valgus)	Low
Conroy (2012)	OA with pain: 170 (59) OA without pain: 91 (58) C:334 (58)	OA with pain: 74.1 ± 3.1 OA without pain: 73.7 ± 2.9 C:73.3 ± 2.7	OA with pain: 30.1 ± 4.8 OA without pain: 30.5 ± 5 C:26.4 ± 4.3	Radiographic and symptomatic	NR	Medial / lateral OA	MUSC (extensor weakness)	High
Fallah-Yakhdani (2012)	OA:16 (68.8) C:12 (58.3)	OA:62.3 ± 10.7 C:62.0 ± 12.6	OA:29.7 ± 4.1 C:29.4 ± 4.9	Symptomatic	NR	Medial / lateral OA	MAL (varus; valgus)	High
Levinger (2012)	OA:32 (50) C:28 (53.6)	OA:65.8 ± 7.5 C:65.2 ± 11.4	OA:29.9 ± 5.2 C:25.5 ± 3.9	Radiographic	NR	Medial OA	LOAD (KADM)	Low
Miyazaki (2012)	mild OA: 20 (100) advanced OA:26 (100) C:22 (100)	mild OA: 3.8 ± 9.2 advanced OA: 74.1 ± 7.9 C:71.8 ± 8.3	mild OA: 22.6 ± 2 advanced OA: 23.5 ± 2.9 C:23.6 ± 2.2	Radiographic	KL2: 20, KL3: 16, KL4: 10	Medial OA	MUSC (extensor weakness); Laxity (anterior-posterior)	Low
Baert (2013a)	OA:24 (100) C:20 (100)	OA:64 ± 7.5 C:62.9 ± 8	OA:27.8 ± 4 C:25.2 ± 3.6	Symptomatic	KL2: 11, KL3: 8, KL4: 5	Medial / lateral OA	MUSC (extensor weakness; flexor weakness); PROP (reposition error)	Low
Baert (2013b)	OA:12 (100) C:14 (100)	OA:68.3 ± 6.8 C:65.8 ± 9.9	OA:28.6 ± 4.1 C: 24.8 ± 3.2	Symptomatic	KL2: 7, KL3: 3, KL4: 2	Medial OA	MAL (varus; valgus); MUSC (extensor weakness)	Low
Kumar (2013)	OA:16 (50) C:12 (50)	OA:65.2 ± 9.5 C:59.5 ± 10.4	OA:28.6 ± 4.3 C:28.4 ± 5.2	Radiographic	NR	Medial OA	MUSC (extensor weakness)	Low
Maly (2013a)^b^	OA:73 (100) C:52 (100)	OA:64.6 ± 6.7 C:60.7 ± 7.1	OA:29.1 ± 4.7 C:26.4 ± 5.0	Radiographic	NR	Medial OA	MUSC (extensor weakness)	Low
Maly (2013b)	OA:31 (29.0) C:30 (53.3)	OA:53.2 ± 6.1 C:33.5 ± 8	OA:30.6 ± 4.3 C:25 ± 4.2	Radiographic and symptomatic	KL2: 14, KL3: 13, KL4: 4	Medial OA	LOAD (KADM)	High
Metcalfe (2013)	OA:20 (45) C:20 (50)	OA:69 ± 7.2 C:68.3 ± 5.9	OA:31.1 ± 3.5 C:26.3 ± 3.6	Radiographic and symptomatic	KL3: 7, KL4: 13	Medial OA	LOAD (KADM)	High
Perry (2013)	OA:19 (52.6) C:17 (47.1)	OA:69.9 ± 6.5 C:66.8 ± 6.4	OA:29.8 ± 3.83 C:28.9 ± 4.7	Symptomatic	NR	Medial / lateral OA	MUSC (extensor weakness)	High
Sagawa (2013)^c^	OA:90 (58.9) C:52 (*n* = 26) (53.8)	OA:68 ± 7 C:66 ± 8	OA:30.4 ± 5.2 C:23.4 ± 2.3	Radiographic and symptomatic	NR	Medial / lateral OA	LOAD (KADM)	Low
Chang (2014)	OA:14 (57) C:14 (57)	OA:60 ± 8.7 C:58.4 ± 9.5	OA:30.5 ± 8 C:23.9 ± 3.1	Radiographic and symptomatic	NR	Medial OA	PROP (TDPM-varus; TDPM-valgus)	Low
Duffell (2014a)	OA:18 (NR) C:18 (NR)	OA:56.4 ± 12.4 C:56.2 ± 13	OA:26.61 C:24.13	Radiographic and symptomatic	NR	Medial OA	LOAD (KADM)	High
Duffell (2014b)	OA:15 (13.3) C:9 (44.4)	OA:54 C:31	OA:25.7 C:22.2	Symptomatic	NR	Medial / lateral OA	MAL (valgus)	High
Favre (2014)	OA:26 (62) C:27 (44)	OA:62 ± 10 C:57 ± 8	OA:29 ± 5 C:27 ± 3	Radiographic and symptomatic	NR	Medial OA	LOAD (KFM; KEM)	Low
Kumar (2014)	OA:30 (50) C:66 (57.6)	OA:57.7 (R:54.3–61.1) C:50.7 (R:48.4–53.1)	OA:26.9 (R:23.5–30.2) C:24.1 (R:23.2–25)	Radiographic and symptomatic	KL2: 10, KL3: 16, KL4: 4	Medial / lateral OA	MUSC (extensor weakness, flexor weakness)	Low
Winters (2014)	OA:26 (46.2) C:23 (47.8)	OA:65.3 ± 7.8 C:63.1 ± 7.7	OA:30.6 ± 4.6 C:25.8 ± 3.8	Radiographic	KL1: 7, KL2: 10, KL3: 10^d, f^	Medial OA	MUSC (extensor weakness); LOAD (KFM; KADM)	High

Legend: *OA* patients with knee osteoarthritis, *C* control group, *NR* not reported, *R* range, *M* median, *SD* standard deviation, *MAL* skeletal malalignment, *MUSC* muscular dysfunction, *PROP* impaired proprioception, *LAX* laxity, *LOAD* abnormal loading, *TDPM* threshold to detect a passive movement, *KFM* knee flexion moment, *KEM* knee extension moment, *KADM* knee adduction moment, *KABM* knee abduction moment, *KIRM* knee internal rotation moment, *KERM* knee external rotation moment, *KL* Kellgren and Lawrence grade. ^a^ This value seems not realistic, but it was stated in the published report, ^b^ Muscle strength was assessed for *n* = 115, ^c^ In controls 52 legs were assessed, ^d^ Patients with KL 1 were included in the osteoarthritis group as selection was based on other inclusion criteria such as pain, ^e^ Number of patients calculated from percentage, ^f^ 27 patients with radiographic severity reported, while 26 osteoarthritis patients are included in the study

As the number of included studies for each specific biomechanical factor was lower than expected, we could not perform all secondary analyses suggested in the protocol. We stratified analyses in the domains malalignment and abnormal loading during gait for medial knee OA, lateral knee OA, and medial/lateral knee OA (a combination of both or involved compartment was not reported), as biomechanical mechanisms will differ based on the involved compartment. The involved compartment was assumed to be medial (or lateral) when 80% or more of the participants was reported to have medial (or lateral) knee OA.

### Assessment of risk of bias of individual studies and overall quality of evidence

Based on the assessment of risk of bias of individual studies, 32 had a low risk of bias, and 27 studies had a high risk of bias (Table 1). High risk of bias was most often based on a high risk in the areas participation and/or outcome measurement. Details regarding each of the six areas of potential study biases can be found in Additional file 3.

### Skeletal malalignment

**Fig. 2:**
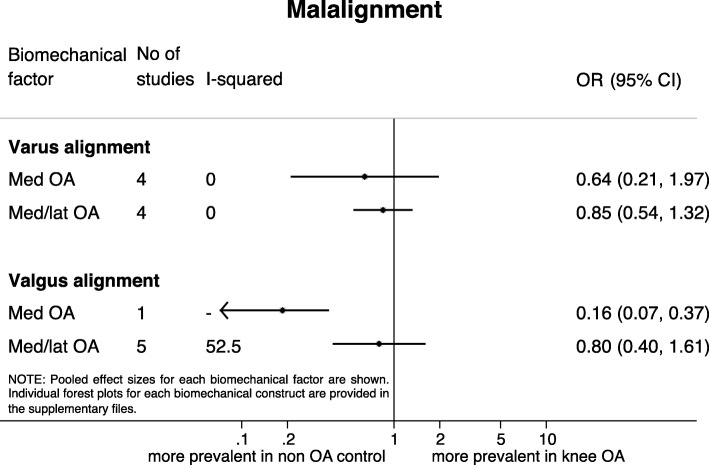
Results of meta-analyses on skeletal malalignment and the presence of knee osteoarthritis. Results stratified for medial knee OA (Med OA) and a combination of both medial and lateral knee OA (or involved compartment not reported (Med/lat OA))

Low quality evidence was found for the odds of having malalignment in patients with knee OA compared to healthy controls (Fig. 2). Varus malalignment, assessed in four studies examining patients with medial knee OA [24–27], and in four studies examining patients with medial and/or lateral knee OA [28–31], was as prevalent in patients with and without knee OA (medial knee OA: OR = 0.64 [95% CI 0.21, 1.97], medial/lateral knee OA: OR = 0.85 [95% CI 0.54, 1.32]). Five studies assessing valgus malalignment in patients with medial and/or lateral knee OA showed no higher odds of having valgus malalignment in patients with knee OA (OR = 0.80 [95% CI 0.40, 1.61]) [28, 30–33]. One study indicated that patients with knee OA have lower odds of having valgus malalignment compared with healthy controls (OR = 0.16 [95% CI 0.07, 0.37]) [24].

### Muscular dysfunction

**Fig. 3:**
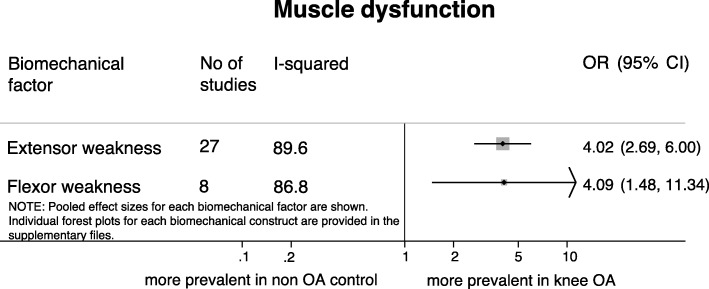
Results of meta-analyses on muscular dysfunction and the presence of knee osteoarthritis

Low quality evidence was found for the odds of having muscle weakness in patients with knee OA compared to controls (Fig. 3). Studies assessing muscle weakness showed that patients with medial and/or lateral knee OA had four times higher odds of having muscle weakness compared with healthy controls, both for extensor [21, 23–25, 27, 34–54] (OR = 4.02 [95% CI 2.69, 6.00], I^2^ = 89.6%, number of studies (k) =27) and flexor muscles [21, 34, 36, 38, 40, 43, 47, 52] (OR = 4.09 [95% CI 1.48, 11.34], I^2^ = 86.8%, k = 8). Both analyses showed considerable heterogeneity. Data regarding neurological characteristics (i.e. co-contraction index) had also been extracted, but differences in outcome measures and measurement techniques made it impossible to combine these.

### Impaired proprioception

**Fig. 4:**
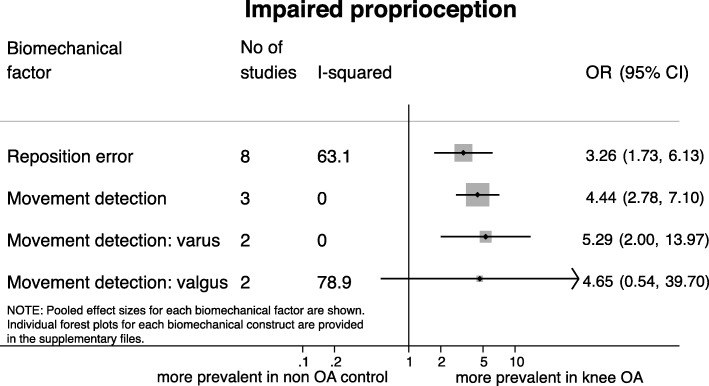
Results of meta-analyses on impaired proprioception and the presence of knee osteoarthritis. Movement detection refers to the threshold to detect a passive movement in the sagittal or frontal plane

Low to moderate quality evidence was found for the odds of having impaired proprioception in patients with knee OA compared to controls (Fig. 4). Eight studies assessing proprioception as reposition error showed that patients with knee OA have higher odds of having higher reposition error (i.e. poorer proprioception) than healthy controls, with substantial heterogeneity (OR = 3.26 [95% CI 1.73, 6.13], I^2^ = 63.1%) [34, 46, 54–59]. The odds of having a higher threshold to detect a passive movement in the sagittal plane was higher in patients with knee OA (OR = 4.44 [95% CI 2.78, 7.10], I^2^ = 0.0%, k = 3), indicating poorer proprioception [57, 60, 62]. Two studies examining knee varus-valgus proprioceptive acuity (i.e. in the frontal plane) showed that patients with knee OA are more likely to have higher threshold to detect a passive movement in the varus direction (OR = 5.29 [95% CI 2.00, 13.97], I^2^ = 0.0%), again indicating poorer proprioception [37, 61]. This was not observed in the valgus direction (OR = 4.65 [95% CI 0.55, 39.70], I^2^ = 78.9%).

### Joint laxity

**Fig. 5:**
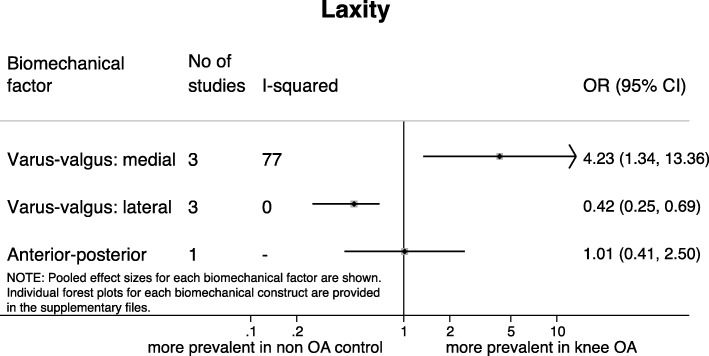
Results of meta-analyses on laxity and the presence of knee osteoarthritis. Varus-valgus laxity is measured at the medial and lateral side of the knee

All studies assessing joint laxity described patients with medial knee OA (Fig. 5). Three studies reported that patients with medial knee OA have four times higher odds of having laxity in varus-valgus direction measured at the medial side of the joint (OR = 4.23 [95% CI 1.34, 13.36], I^2^ = 77.0%) [22, 27, 63], however, evidence was of low quality and considerable heterogeneity was present. The same three studies reported that patients with medial knee OA have lower odds of having varus-valgus laxity measured at the lateral side of the joint (OR = 0.42 [95% CI 0.25, 0.69], I^2^ = 0.0%). Evidence was of low quality. The only study that assessed laxity in anterior-posterior direction suggested no higher or lower odds for patients with medial knee OA compared to healthy controls [23].

### Abnormal loading during gait

**Fig. 6:**
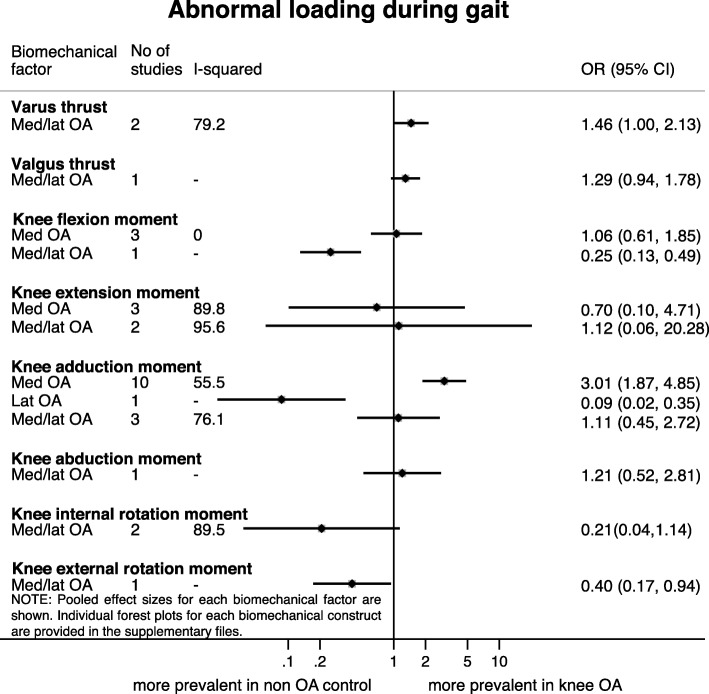
Results of meta-analyses on abnormal loading during gait and the presence of knee osteoarthritis. Results stratified for medial knee OA (Med OA), lateral knee OA (Lat OA), and a combination of both medial and lateral knee OA (or involved compartment not reported (Med/lat OA))

Very low to low quality evidence was found for most odds of having abnormal loading in patients with medial and/or lateral knee OA, only moderate quality evidence was found for the odds of having a higher KADM in patients with medial knee OA (Fig. 6). The two studies examining thrust concerned a population with medial and/or lateral knee OA. The odds of having varus thrust was higher in knee OA patients (OR = 1.46 [95% CI 1.00, 2.13], I^2^ = 79.2%) [64, 65], while patients with knee OA had no higher odds of having valgus thrust [65]. The odds of having a higher KFM was not higher in patients with medial knee OA [27, 50, 67], while significant higher odds were found for a higher KFM in the healthy controls in one study compared with patients with medial/lateral knee OA [66]. The odds for the presence of a higher knee extension moment (KEM) were not higher in patients with medial and/or lateral knee OA, with considerable heterogeneity [27, 67–70]. Ten studies showed that patients with medial knee OA had higher odds of having a higher knee adduction moment (KADM) (OR = 3.01 [95% CI 1.87, 4.85], I^2^ = 55.5%) [25, 27, 50, 71–75, 77, 78]. The only study [71] assessing this in patients with lateral knee OA reported higher odds for having higher KADM values in healthy controls, and patients with medial/lateral knee OA had no higher or lower odds than healthy controls (OR = 1.11 [95% CI 0.45, 2.72], I^2^ = 76.1%, k = 3) [69, 70, 76]. One study assessing both knee abduction moment (KABM) and knee external rotation moment (KERM) showed only higher odds in patients with medial/lateral knee OA for having a higher KERM [70]. The odds for having a higher knee internal rotation moment (KIRM) was not higher in patients with medial and/or lateral knee OA compared to healthy controls (OR = 0.21 [95% CI 0.04, 1.14], I^2^ = 89.5%, k = 2) [66, 70].

## Discussion

This systematic review and meta-analyses aimed at investigating the association of specific biomechanical factors with knee OA and knee OA development, and potential effect modification of other relevant risk factors on this association. Based on the predefined selection criteria we did not include any longitudinal studies and sparse data combined with variations in studies for most meta-analyses made precluding any assessment of other relevant risk factors. As a result we could not investigate the association between biomechanical factors and knee OA development, and the impact of other relevant risk factors on any such associations. Thus, this study provides a comprehensive overview of the direction and magnitude of associations between different biomechanical factors and presence of knee OA. Based on mainly low quality evidence following GRADE, the results from this review indicate that patients with knee OA are characterized by lower knee extensor/flexor muscle strength, proprioception deficits, more medial varus-valgus laxity, less lateral varus-valgus laxity, and more varus thrust than healthy controls. Further, patients with medial knee OA are more likely to have a high knee adduction moment compared with healthy controls.

We intended to include longitudinal cohort studies to assess biomechanical risk factors for development of knee OA. However, based on our relative strict inclusion criteria allowing only studies where healthy individuals were available as comparators for assessing biomechanical risk factors, we were not able to include any cohort studies in this review. This was decided because changes in one leg may potentially impact the contralateral leg [4, 57, 79].

Impaired biomechanics is considered to be an important component of knee OA and such characteristics are often the justification of specific research questions. Although there is considerable evidence from individual studies describing those factors, we could not identify any attempts to summarize the association of skeletal malalignment, muscular dysfunction, impaired proprioception and laxity with knee OA by providing pooled estimates of associations. Pooled estimates have only been presented for the association of abnormal loading with knee OA [12]. This study expands this knowledge by providing an overview of the evidence of which biomechanical attributes describe patients with knee OA.

Our findings that decreased extensor/flexor strength and impaired proprioception are associated with knee OA confirm results of earlier narrative reviews [4–6, 80]. In addition, Freisinger et al. also found that patients with medial knee OA have an increased medial, but not lateral laxity [7]. Biomechanical characteristics during level walking have been the subject of a systematic review and meta-analysis by Mills et al. [12]. In agreement with our study, Mills reported conflicting evidence for the association of KFM with knee OA, which might be due to adaptations of walking pattern to reduce pain and instability [81]. In the same study, evidence for an association of KADM with knee OA was found to be inconclusive, whereas we found that patients with medial knee osteoarthritis have a higher KADM. Lack of stratification for the involved joint compartment in the study by Mills and co-workers could influence the results, as biomechanical mechanisms are expected to differ depending on the involved compartment. We were not able to stratify our varus and valgus thrust analysis for the involved compartment. In combination with the low number of included studies for those factors, this might explain why we only found a borderline significant association for varus thrust with knee OA.

Skeletal malalignment is also commonly reported to be present in patients with knee OA. Surprisingly, this was not found in the present study, although evidence was of low quality and methods used to assess malalignment were not uniform. Several individual studies have reported that varus and/or valgus malalignment are risk factors for the development and/or progression of knee OA [82–84], although one study stated that malalignment is not associated with knee OA development, and suggested that it rather is a marker of disease severity or its progression [85]. Those studies were not included in this review. The main reason for excluding studies was that they compared between knees instead of persons (i.e. allowing the contra-lateral leg as control), as evidence suggests that biomechanical factors are also altered in the contralateral knee highlighting the importance of an independent comparator [4, 57]. Another approach for future attempts to summarize such evidence, could be to include all available data and investigate the importance of type of control (i.e. healthy controls or contra-lateral leg) in sensitivity analysis to also be able to include more longitudinal studies. A systematic review and meta-analysis showed limited evidence for an association between knee malalignment and incident knee OA, although it also showed a relationship between varus and valgus alignment and structural progression of knee OA [8].

Limitations of this study warrant consideration. Our findings should be interpreted with caution due to the small number of included studies and the small sample size for most of the biomechanical factors (in particular skeletal malalignment and laxity). As a consequence of the above, and because all studies had a cross-sectional design, we rated most of the quality of evidence to low. Future studies should aim to improve the quality of the evidence.

This systematic review and meta-analysis identified several biomechanical characteristics of patients with knee OA. These findings are important for clinicians, as identification of such biomechanical impairments may help clinicians to better tailor interventions to the individual patient. In fact, the specific biomechanical profile of one patient may mean that some biomechanical interventions could be beneficial and others ineffective or even harmful. Clinical practices may not have the necessary specialized equipment to perform the biomechanical measurements that were performed in the included studies. However, clinical proxy measures exist to examine many of these biomechanical impairments (e.g. manual assessment of laxity or visual assessment of loading during gait by varus or valgus thrust). In addition, longitudinal cohort studies are needed to evaluate the importance of biomechanical factors in the development of knee osteoarthritis. Those studies should aim to evaluate biomechanical factors as risk factors for the development of knee OA. Furthermore, they should focus on the identification of subgroups. This will facilitate identification of persons at high risk of developing knee OA, who might want to be engaged in prevention programs. The longitudinal studies should include healthy persons, who have general risk factors for the development of knee OA, but do not have knee OA at baseline. The presence of knee OA should be assessed at baseline and follow up. General risk factors [86] and at least the biomechanical factors we identified as knee OA characteristics should be assessed. Data of healthy individuals should be compared with data of individuals developing knee OA in one or both knees.

## Conclusions

In conclusion, results indicate that patients with knee OA are more likely to display a number of biomechanical characteristics such as lower muscle strength, proprioception deficits, more medial varus-valgus laxity and less lateral varus-valgus laxity and higher knee adduction moment (medial knee OA only) compared with healthy controls. The causal relationship between biomechanical factors and the development of knee OA could not be determined as no longitudinal studies were included. High quality longitudinal studies are needed to evaluate the impact of biomechanical factors on the development of knee OA.

## Additional files

Additional file 1: Summary of findings table (DOCX 71 kb).

Additional file 2: Forest plots of data pooling for skeletal malalignment, muscular dysfunction, impaired proprioception, laxity and abnormal loading, and the presence of knee osteoarthritis (ZIP 1272 kb).

Additional file 3: Risk of bias and individual study quality for the included studies (DOCX 73 kb).

## Abbreviations

BMI: Body mass index
C: Control group
CI: Confidence interval
GRADE: Grading of Recommendation, Assessment, Development and Evaluation
k: Number of studies
KABM: Knee abduction moment
KADM: Knee adduction moment
KEM: Knee extension moment
KERM: Knee external rotation moment
KFM: Knee flexion moment
KIRM: Knee internal rotation moment
KL: Kellgren and Lawrence grade
LAX: Laxity
LOAD: Abnormal loading
M: Median
MAL: Skeletal malalignment
MUSC: Muscular dysfunction
n: Number of persons
NR: Not reported
OA: Osteoarthritis
OR: Odds ratio
PRISMA: Preferred Reporting Items for Systematic Reviews and Meta-analyses
PROP: Impaired proprioception
QUIPS tool: Quality In Prognostic Studies tool
R: Range
SD: Standard deviation
TDPM: Threshold to detect a passive movement
