# Supplementary material for: Association of malalignment, muscular dysfunction, proprioception, laxity and abnormal joint loading with tibiofemoral knee osteoarthritis - a systematic review and meta-analysis
Source: BMC Musculoskelet Disord. 2018 Jul 28;19:273. doi: 10.1186/s12891-018-2202-8 (PMC6064629; doi:10.1186/s12891-018-2202-8)
Supplement: Supplementary file 1 — Summary of findings table (DOCX 71 kb). [file 12891_2018_2202_MOESM1_ESM.docx]

**ADDITIONAL FILE 1. Summary of findings table**

**The basis for the assumed risk is provided in footnotes**

OR = Odds ratio, CI = confidence interval, OA = knee osteoarthritis, KADM = knee adduction moment, KABM = knee abduction moment, KFM = knee flexion moment, KEM = knee extension moment, KIRM = knee internal rotation moment, KERM = knee external rotation moment.

**GRADE Working Group grades of evidence**

**High quality:** Further research is very unlikely to change our confidence in the estimate of effect.

**Moderate quality:** Further research is likely to have an important impact on our confidence in the estimate of effect and may change the estimate.

**Low quality:** Further research is very likely to have an important impact on our confidence in the estimate of effect and is likely to change the estimate.

**Very low quality:** We are very uncertain about the estimate.

^a^ Observational studies start at low quality of evidence

^b^ Downgraded due to inconsistency.

^c^ Downgraded due to indirectness.

^d^ Downgraded due to sparse data.

^e^ Upgraded due to high association.

| **Biomechanical factor** | **Analyses stratified for** | **No of participants (studies)** | **Pooled OR (95% CI)** | **I-squared** | **Level of evidence** ^a^ |
| --- | --- | --- | --- | --- | --- |
| **Skeletal malalignment** | | | | | |
| Varus | Medial OA | 144 (4) | 0.64 (0.21, 1.97) | 0 | Low |
|  | Medial/lateral OA | 681 (4) | 0.85 (0.54, 1.32) | 0 | Low |
| Valgus | Medial OA | 26 (1) | 0.16 (0.07, 0.37) | - | Low |
|  | Medial/lateral OA | 700 (5) | 0.80 (0.40, 1.61) | 52.5 | Low |
| **Muscular dysfunction** |  |  |  |  |  |
| Extensor weakness |  | 6086 (27) | 4.02 (2.69, 6.00) | 89.6 | Low ^b, e^ |
| Flexor weakness |  | 547 (8) | 4.09 (1.48, 11.34) | 86.8 | Low ^b, e^ |
| **Proprioception** | | | | | |
| Reposition error |  | 394 (8) | 3.26 (1.73, 6.13) | 63.1 | Low ^b, e^ |
| Threshold to detect a passive movement (sagittal plane) |  | 166 (3) | 4.44 (2.78, 7.10) | 0 | Moderate ^e^ |
| Threshold to detect a passive movement (Varus direction) |  | 55 (2) | 5.29 (2.00, 13.97) | 0 | Moderate ^e^ |
| Threshold to detect a passive movement (Valgus direction) |  | 55 (2) | 4.65 (0.54, 39.70) | 78.9 | Low ^b, e^ |
| **Laxity** | | | | | |
| Valgus-varus; measured at medial side |  | 253 (3) | 4.23 (1.34, 13.36) | 77 | Low ^b, e^ |
| Valgus-varus; measured at lateral side |  | 253 (3) | 0.42 (0.25, 0.69) | 0 | Low |
| Anterior-posterior |  | 68 (1) | 1.01 (0.41, 2.50) | - | Low |
| **Abnormal loading during gait** | | | | | |
| Varus thrust | Medial/lateral OA | 4848 (2) | 1.46 (1.00, 2.13) | 79.2 | Low |
| Valgus thrust | Medial/lateral OA | 3592 (1) | 1.29 (0.94, 1.78) | - | Low |
| KFM | Medial OA | 156 (3) | 1.06 (0.61, 1.85) | 0 | Low |
|  | Medial/lateral OA | 121 (1) | 0.25 (0.13, 0.49) | - | Low |
| KEM | Medial OA | 155 (3) | 0.70 (0.10, 4.71) | 89.8 | Very low ^b^ |
|  | Medial/lateral OA | 240 (2) | 1.12 (0.06, 20.28) | 95.6 | Very low ^b^ |
| KADM | Medial OA | 548 (10) | 3.01 (1.87, 4.85) | 55.5 | Moderate ^e^ |
|  | Lateral OA | 45 (1) | 0.09 (0.02, 0.35) | - | Low ^d, e^ |
|  | Medial/lateral OA | 356 (3) | 1.11 (0.45, 2.72) | 76.1 | Very low ^b^ |
| KABM | Medial/lateral OA | 159 (1) | 1.21 (0.52, 2.81) | - | Low |
| KIRM | Medial/lateral OA | 280 (2) | 0.21 (0.04, 1.14) | 89.5 | Low ^b, e^ |
| KERM | Medial/lateral OA | 159 (1) | 0.40 (0.17, 0.94) | - | Low |
