# Supplementary material for: Association of malalignment, muscular dysfunction, proprioception, laxity and abnormal joint loading with tibiofemoral knee osteoarthritis - a systematic review and meta-analysis
Source: BMC Musculoskelet Disord. 2018 Jul 28;19:273. doi: 10.1186/s12891-018-2202-8 (PMC6064629; doi:10.1186/s12891-018-2202-8)
Supplement: Supplementary file 2 — Forest plots of data pooling for skeletal malalignment, muscular dysfunction, impaired proprioception, laxity and abnormal loading, and the presence of knee osteoarthritis (ZIP 1272 kb). [file 12891_2018_2202_MOESM2_ESM.zip › Additional file 2. Forest plotsR1.docx]

**ADDITIONAL FILE 2.**

Forest plots of data pooling for skeletal malalignment, muscular dysfunction, impaired proprioception, laxity and abnormal loading, and the presence of knee osteoarthritis.

OR = Odds ratio, CI = confidence interval, OA = knee osteoarthritis.

*Skeletal malalignment*

**ADD. FILE 2 FIGURE 1 HERE** (varus malalignment)

**ADD. FILE 2 FIGURE 2 HERE** (valgus malalignment)

*Muscular dysfunction*

**ADD. FILE 2 FIGURE 3 HERE** (extensor weakness)

**ADD. FILE 2 FIGURE 4 HERE** (flexor weakness)

*Impaired proprioception*

**ADD. FILE 2 FIGURE 5 HERE** (reposition error)

**ADD. FILE 2 FIGURE 6 HERE** (threshold to detect passive moment – sagittal plane)

**ADD. FILE 2 FIGURE 7 HERE** (threshold to detect passive moment – frontal plane – varus direction)

**ADD. FILE 2 FIGURE 8 HERE** (threshold to detect passive moment – frontal plane – valgus direction)

*Laxity*

**ADD. FILE 2 FIGURE 9 HERE** (varus-valgus laxity measured at medial side)

**ADD. FILE 2 FIGURE 10 HERE** (varus-valgus laxity measured at lateral side)

**ADD. FILE 2 FIGURE 11 HERE** (anterior-posterior laxity)

*Abnormal loading during gait*

**ADD. FILE 2 FIGURE 12 HERE** (varus thrust)

**ADD. FILE 2 FIGURE 13 HERE** (valgus thrust)

**ADD. FILE 2 FIGURE 14 HERE** (knee flexion moment)

**ADD. FILE 2 FIGURE 15 HERE** (knee extension moment)

**ADD. FILE 2 FIGURE 16 HERE** (knee adduction moment)

**ADD. FILE 2 FIGURE 17 HERE** (knee abduction moment)

**ADD. FILE 2 FIGURE 18 HERE** (knee internal rotation moment)

**ADD. FILE 2 FIGURE 19 HERE** (knee external rotation moment)
