# Supplementary figures and images for: Association of malalignment, muscular dysfunction, proprioception, laxity and abnormal joint loading with tibiofemoral knee osteoarthritis - a systematic review and meta-analysis
Source: BMC Musculoskelet Disord. 2018 Jul 28;19:273. doi: 10.1186/s12891-018-2202-8 (PMC6064629; doi:10.1186/s12891-018-2202-8)

# Varus malalignment

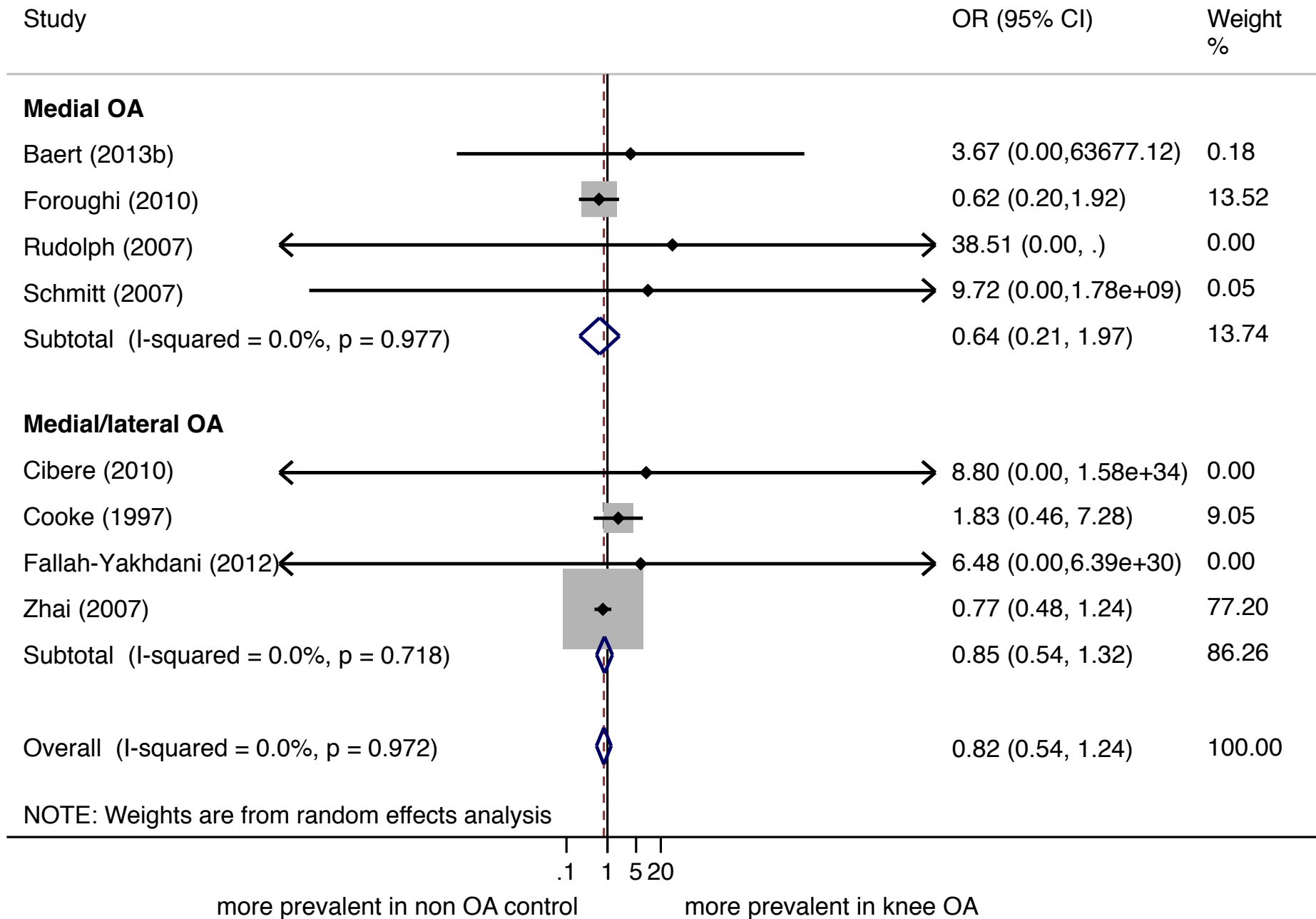

Supplement: Supplementary file 2 — Forest plots of data pooling for skeletal malalignment, muscular dysfunction, impaired proprioception, laxity and abnormal loading, and the presence of knee osteoarthritis (ZIP 1272 kb). [file 12891_2018_2202_MOESM2_ESM.zip › Add. file 2 figure 1 varus malalignmentR1.pdf]

# Varus-valgus laxity measured at lateral side

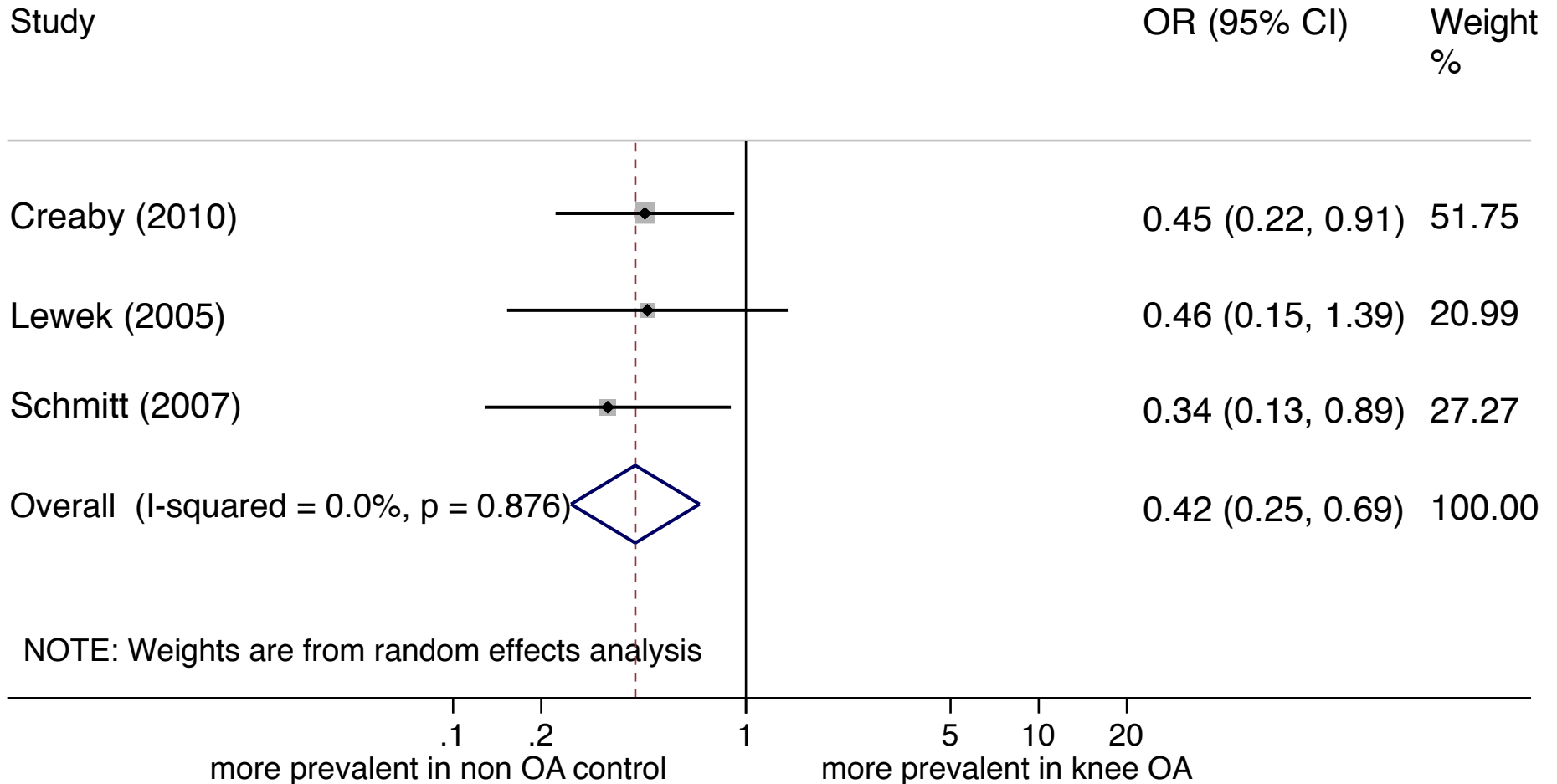

Supplement: Supplementary file 2 — Forest plots of data pooling for skeletal malalignment, muscular dysfunction, impaired proprioception, laxity and abnormal loading, and the presence of knee osteoarthritis (ZIP 1272 kb). [file 12891_2018_2202_MOESM2_ESM.zip › Add. file 2 figure 10 varus-valgus laxity lateralR1.pdf]

# Anterior-posterior laxity

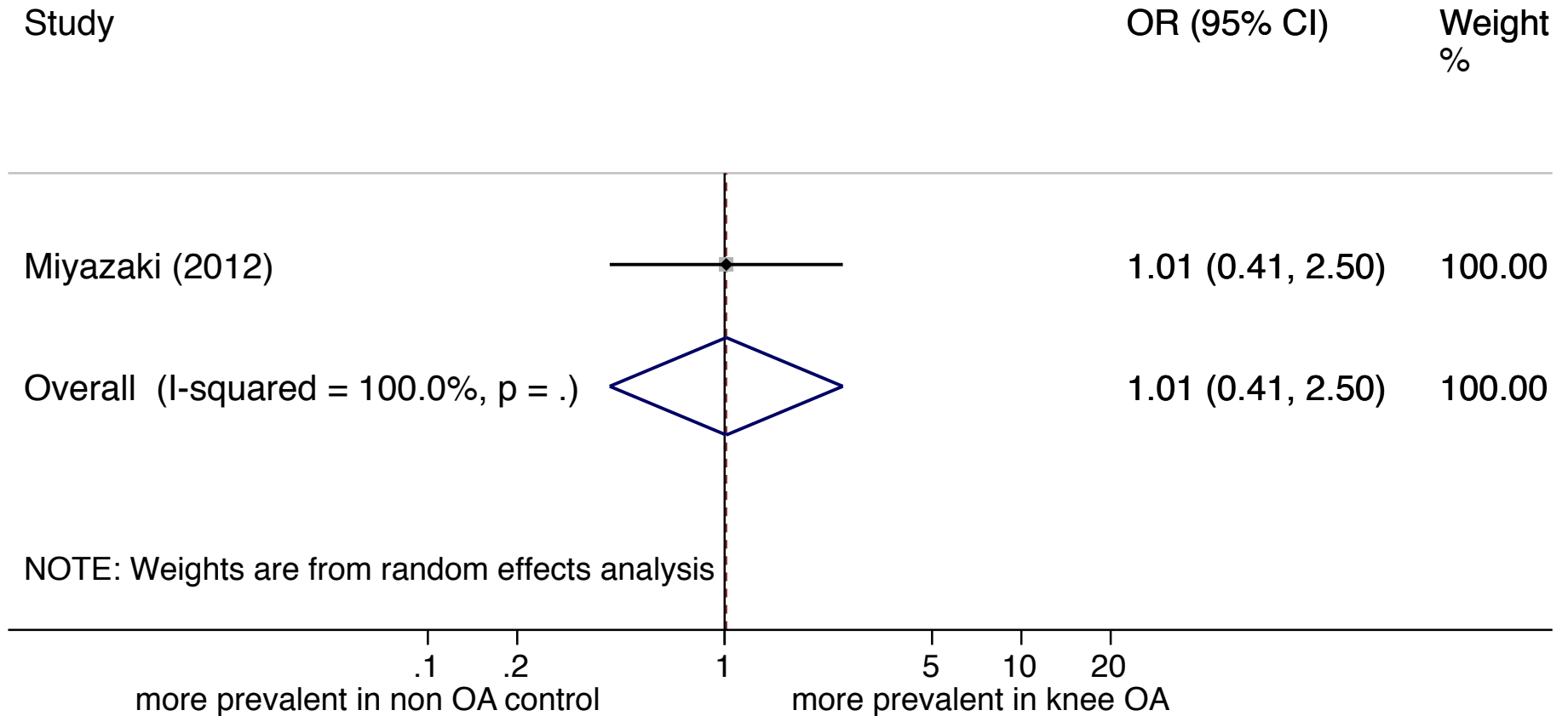

Supplement: Supplementary file 2 — Forest plots of data pooling for skeletal malalignment, muscular dysfunction, impaired proprioception, laxity and abnormal loading, and the presence of knee osteoarthritis (ZIP 1272 kb). [file 12891_2018_2202_MOESM2_ESM.zip › Add. file 2 figure 11 anterior-posterior laxityR1.pdf]

# Varus thrust

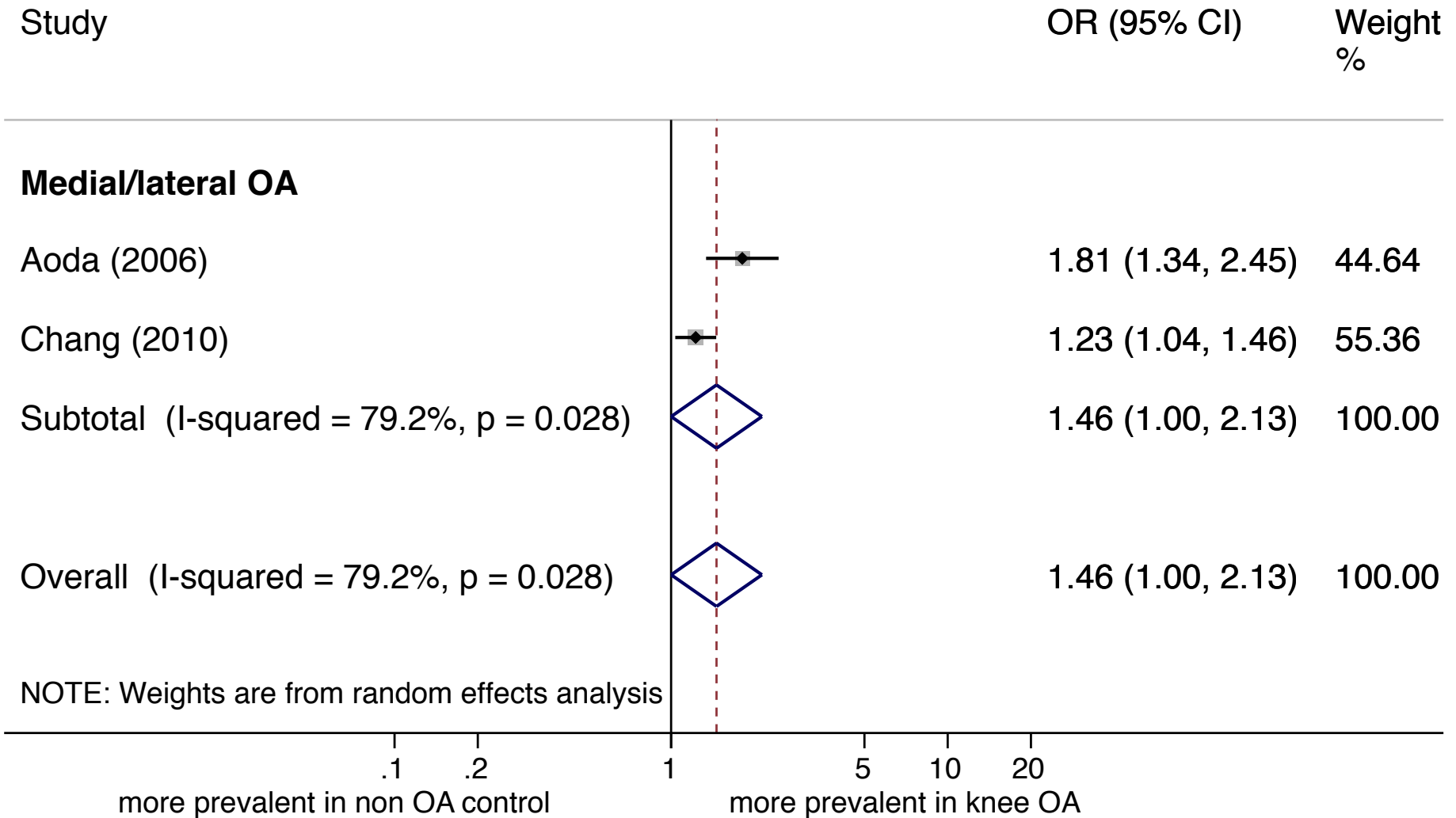

Supplement: Supplementary file 2 — Forest plots of data pooling for skeletal malalignment, muscular dysfunction, impaired proprioception, laxity and abnormal loading, and the presence of knee osteoarthritis (ZIP 1272 kb). [file 12891_2018_2202_MOESM2_ESM.zip › Add. file 2 figure 12 varus thrustR1.pdf]

# Valgus thrust

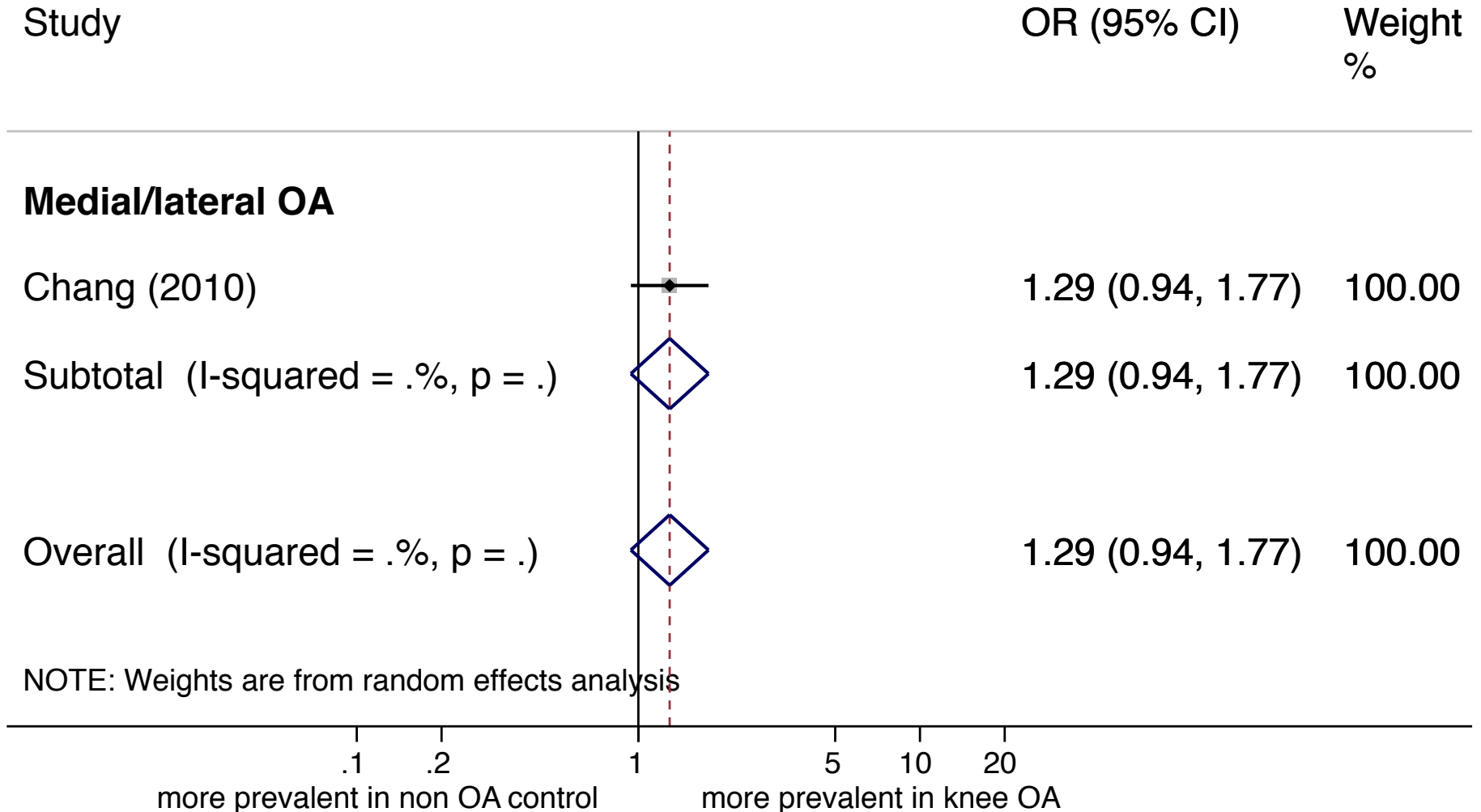

Supplement: Supplementary file 2 — Forest plots of data pooling for skeletal malalignment, muscular dysfunction, impaired proprioception, laxity and abnormal loading, and the presence of knee osteoarthritis (ZIP 1272 kb). [file 12891_2018_2202_MOESM2_ESM.zip › Add. file 2 figure 13 valgus thrustR1.pdf]

# Knee flexion moment

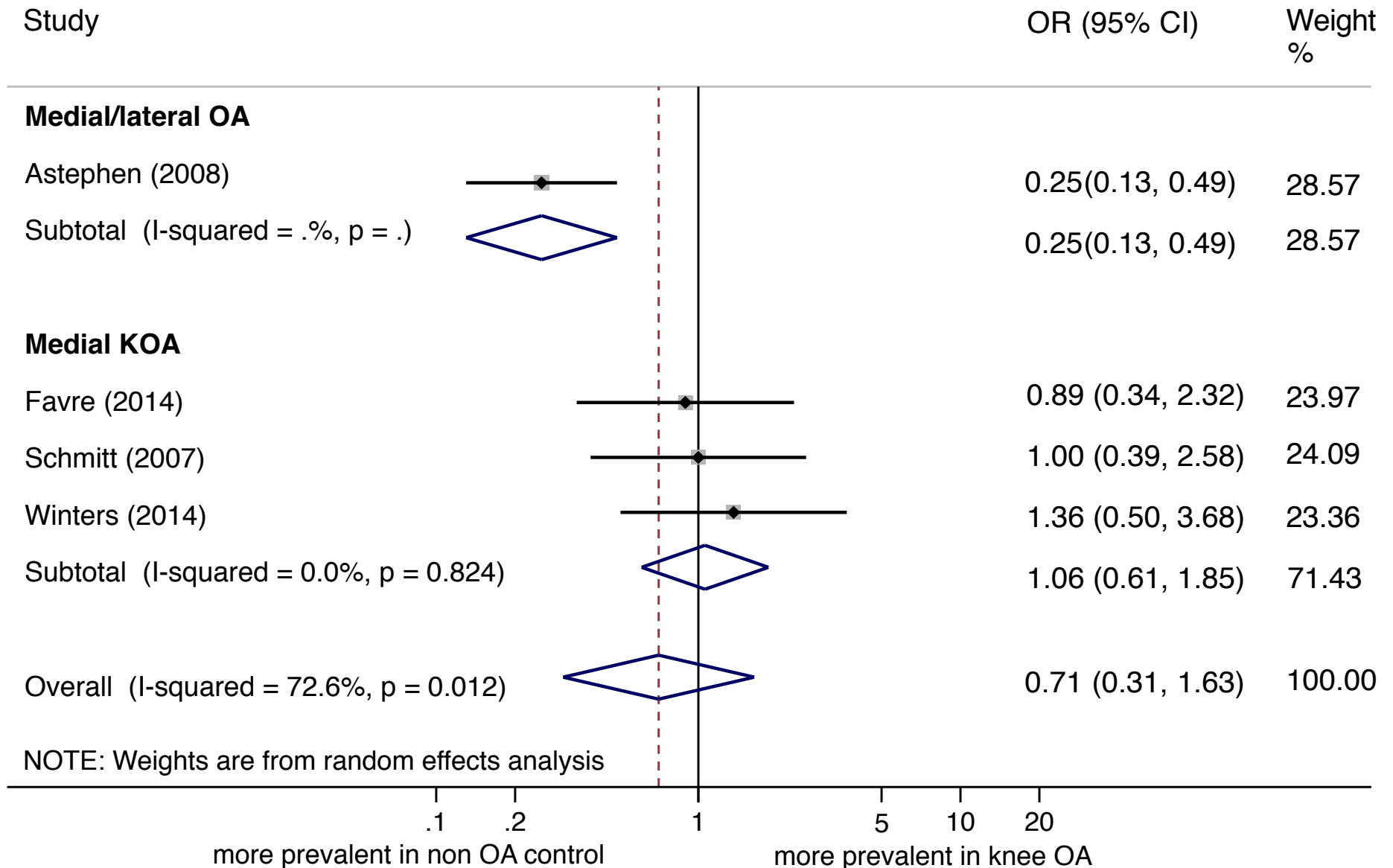

Supplement: Supplementary file 2 — Forest plots of data pooling for skeletal malalignment, muscular dysfunction, impaired proprioception, laxity and abnormal loading, and the presence of knee osteoarthritis (ZIP 1272 kb). [file 12891_2018_2202_MOESM2_ESM.zip › Add. file 2 figure 14 KFMR1.pdf]

# Knee extension moment

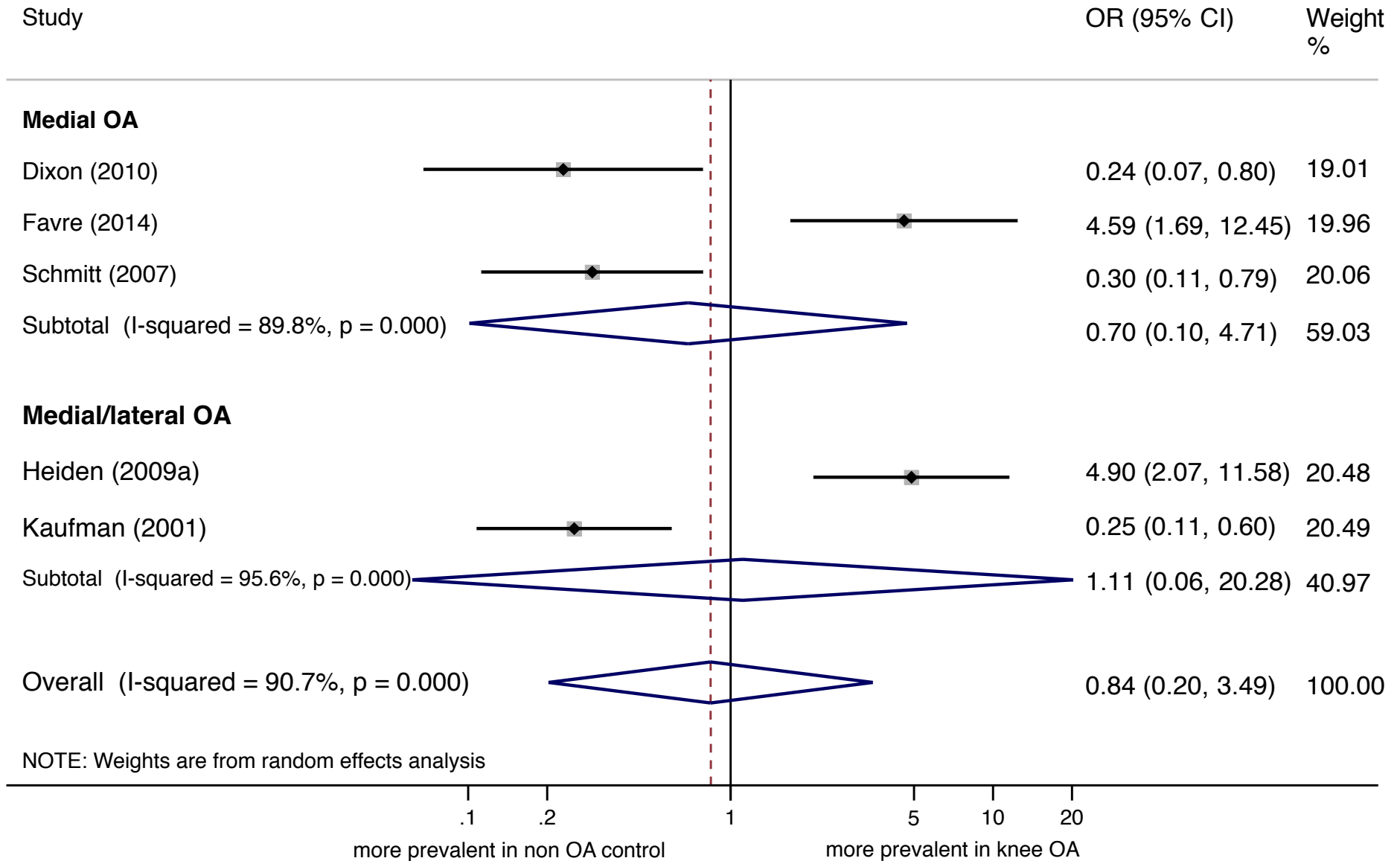

Supplement: Supplementary file 2 — Forest plots of data pooling for skeletal malalignment, muscular dysfunction, impaired proprioception, laxity and abnormal loading, and the presence of knee osteoarthritis (ZIP 1272 kb). [file 12891_2018_2202_MOESM2_ESM.zip › Add. file 2 figure 15 KEMR1.pdf]

# Knee adduction moment

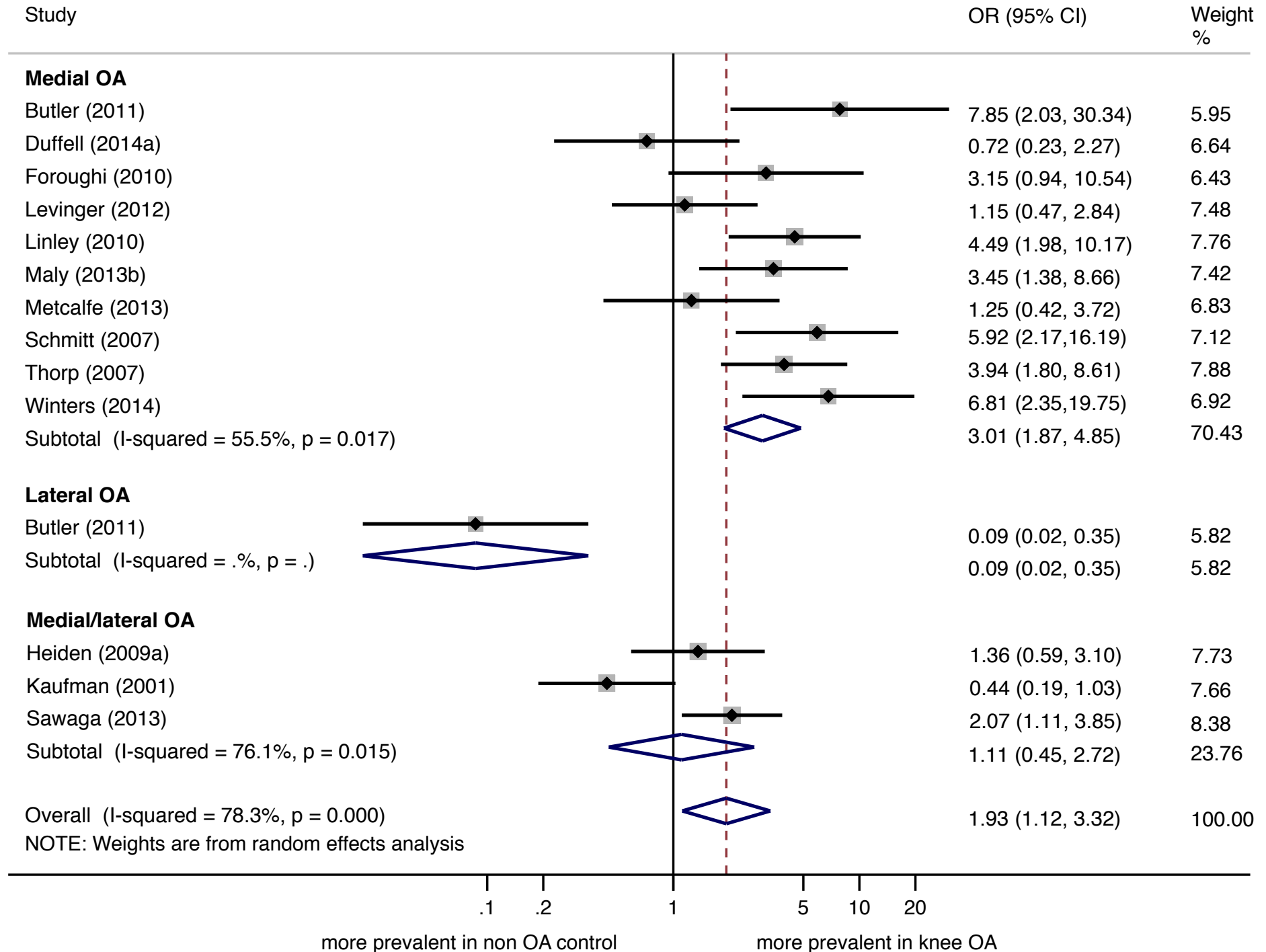

Supplement: Supplementary file 2 — Forest plots of data pooling for skeletal malalignment, muscular dysfunction, impaired proprioception, laxity and abnormal loading, and the presence of knee osteoarthritis (ZIP 1272 kb). [file 12891_2018_2202_MOESM2_ESM.zip › Add. file 2 figure 16 KADMR1.pdf]

# Knee abduction moment

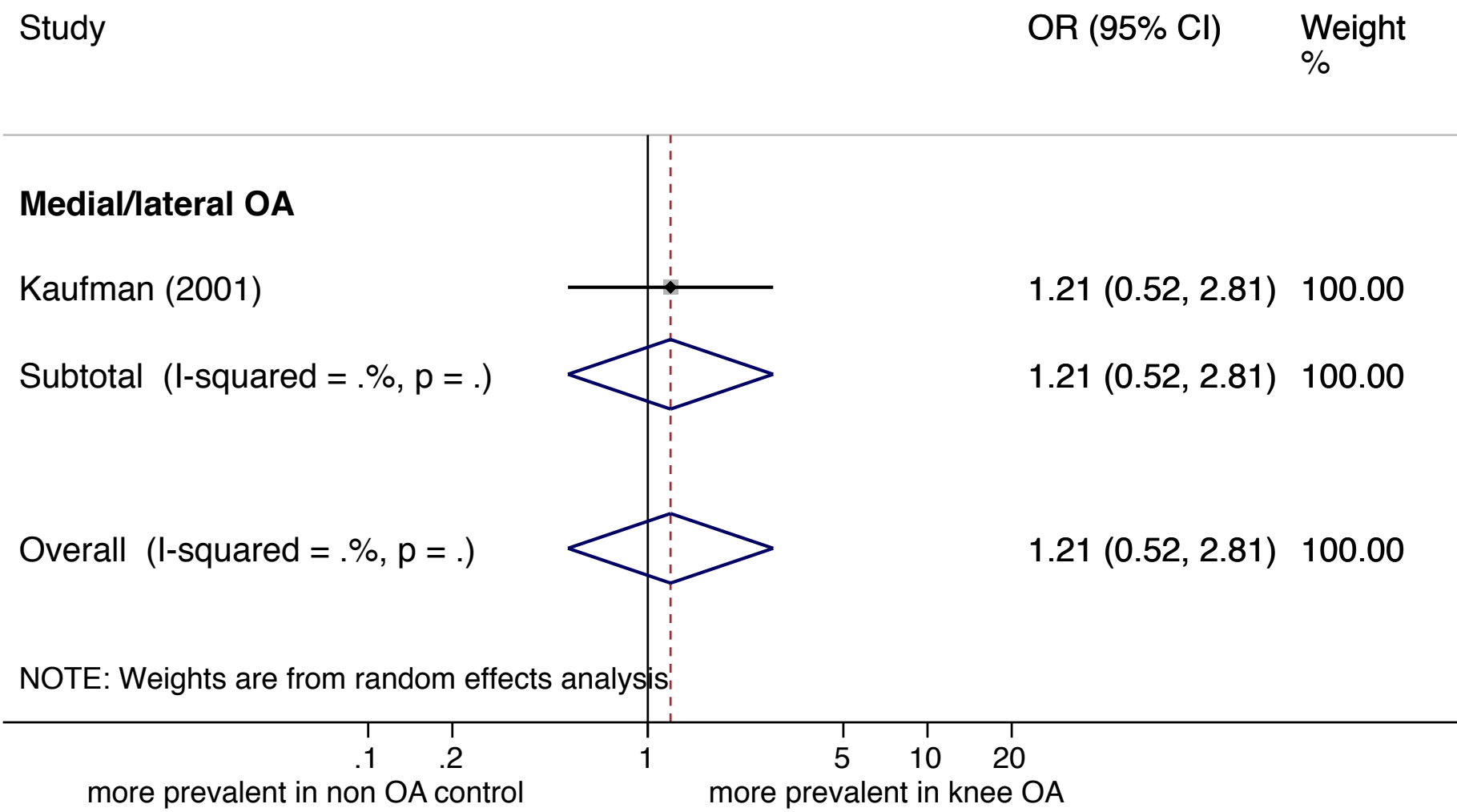

Supplement: Supplementary file 2 — Forest plots of data pooling for skeletal malalignment, muscular dysfunction, impaired proprioception, laxity and abnormal loading, and the presence of knee osteoarthritis (ZIP 1272 kb). [file 12891_2018_2202_MOESM2_ESM.zip › Add. file 2 figure 17 KABMR1.pdf]

# Knee internal rotation moment

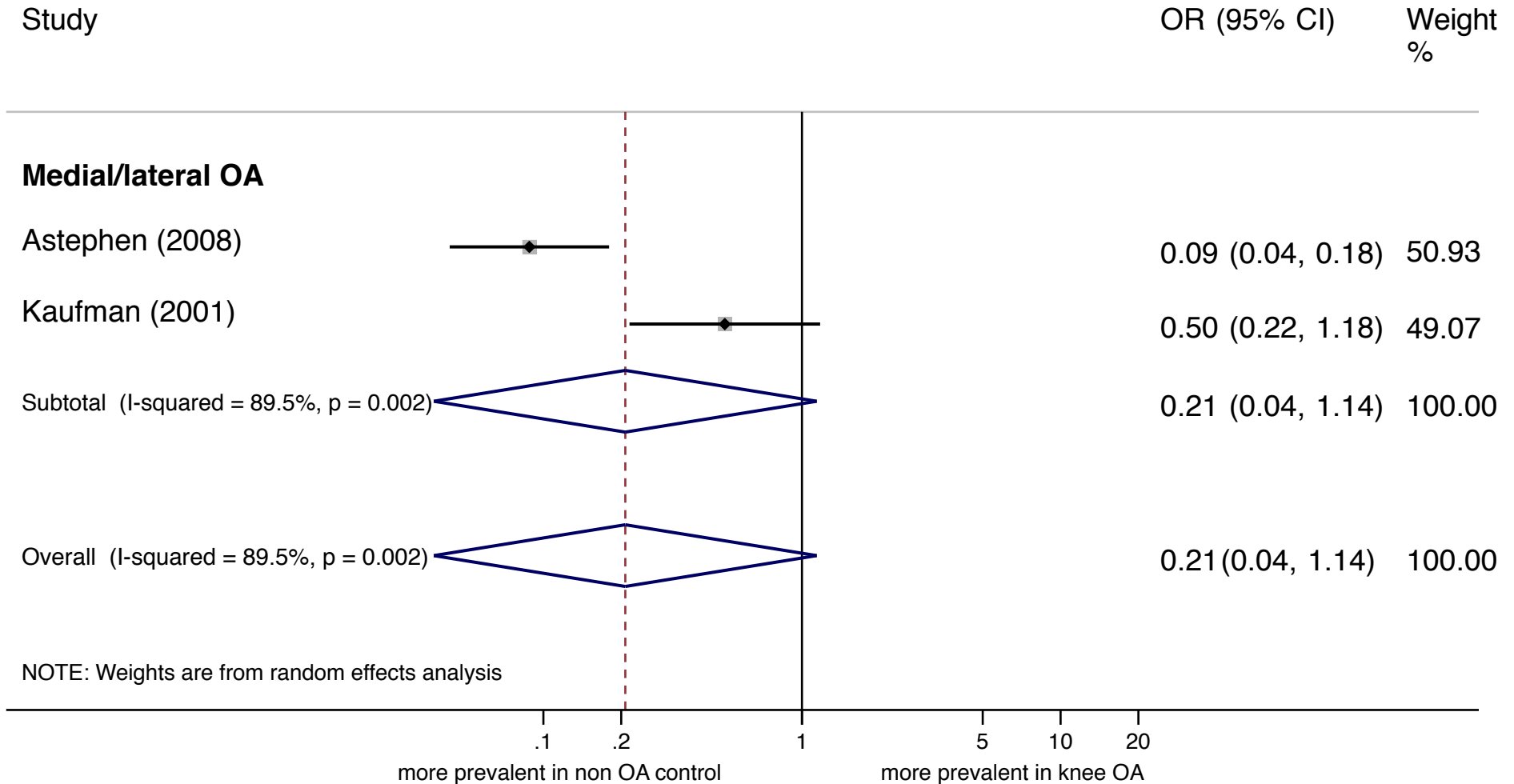

Supplement: Supplementary file 2 — Forest plots of data pooling for skeletal malalignment, muscular dysfunction, impaired proprioception, laxity and abnormal loading, and the presence of knee osteoarthritis (ZIP 1272 kb). [file 12891_2018_2202_MOESM2_ESM.zip › Add. file 2 figure 18 KIRMR1.pdf]

# Knee external rotation moment

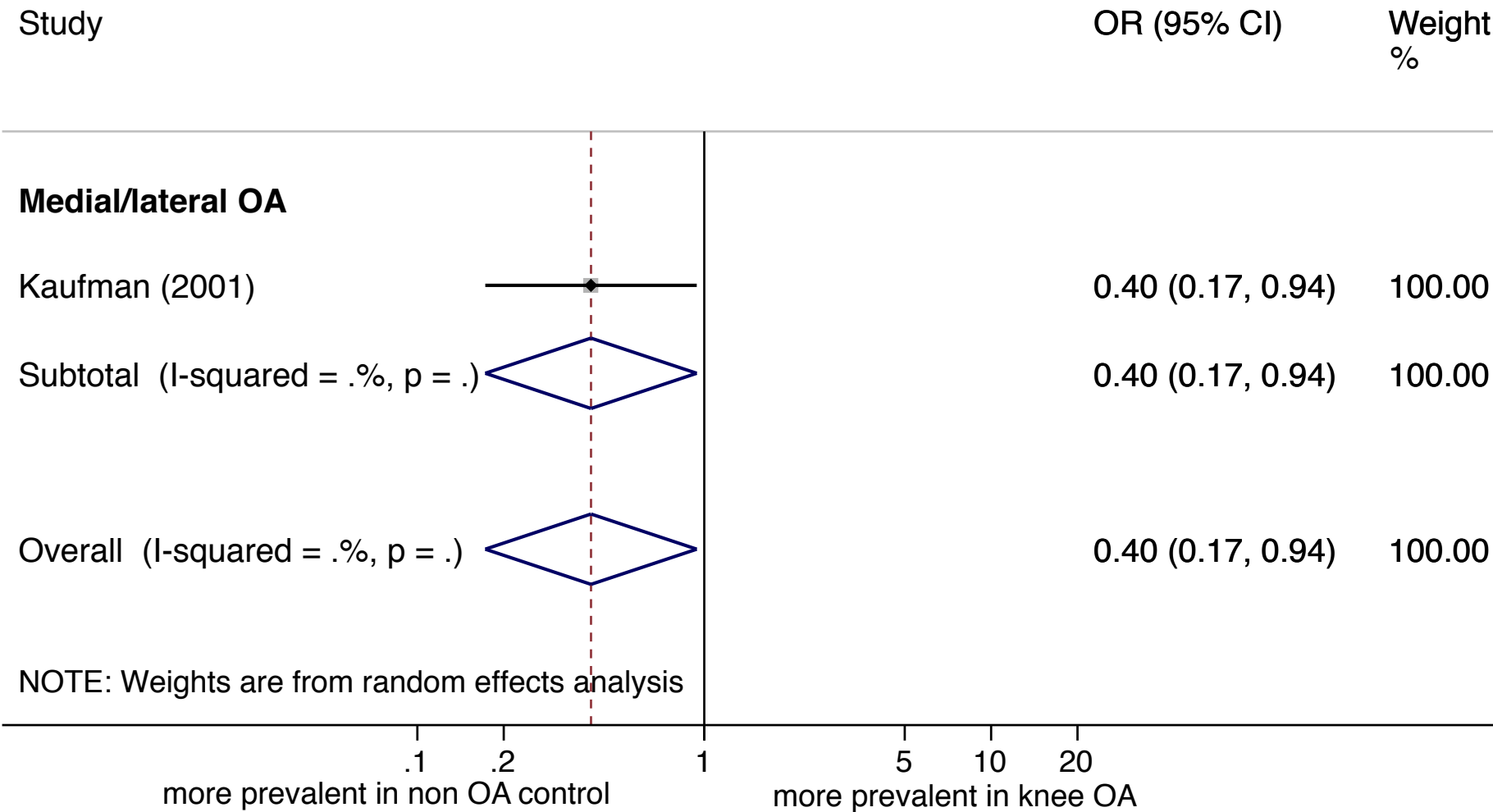

Supplement: Supplementary file 2 — Forest plots of data pooling for skeletal malalignment, muscular dysfunction, impaired proprioception, laxity and abnormal loading, and the presence of knee osteoarthritis (ZIP 1272 kb). [file 12891_2018_2202_MOESM2_ESM.zip › Add. file 2 figure 19 KERMR1.pdf]

# Valgus malalignment

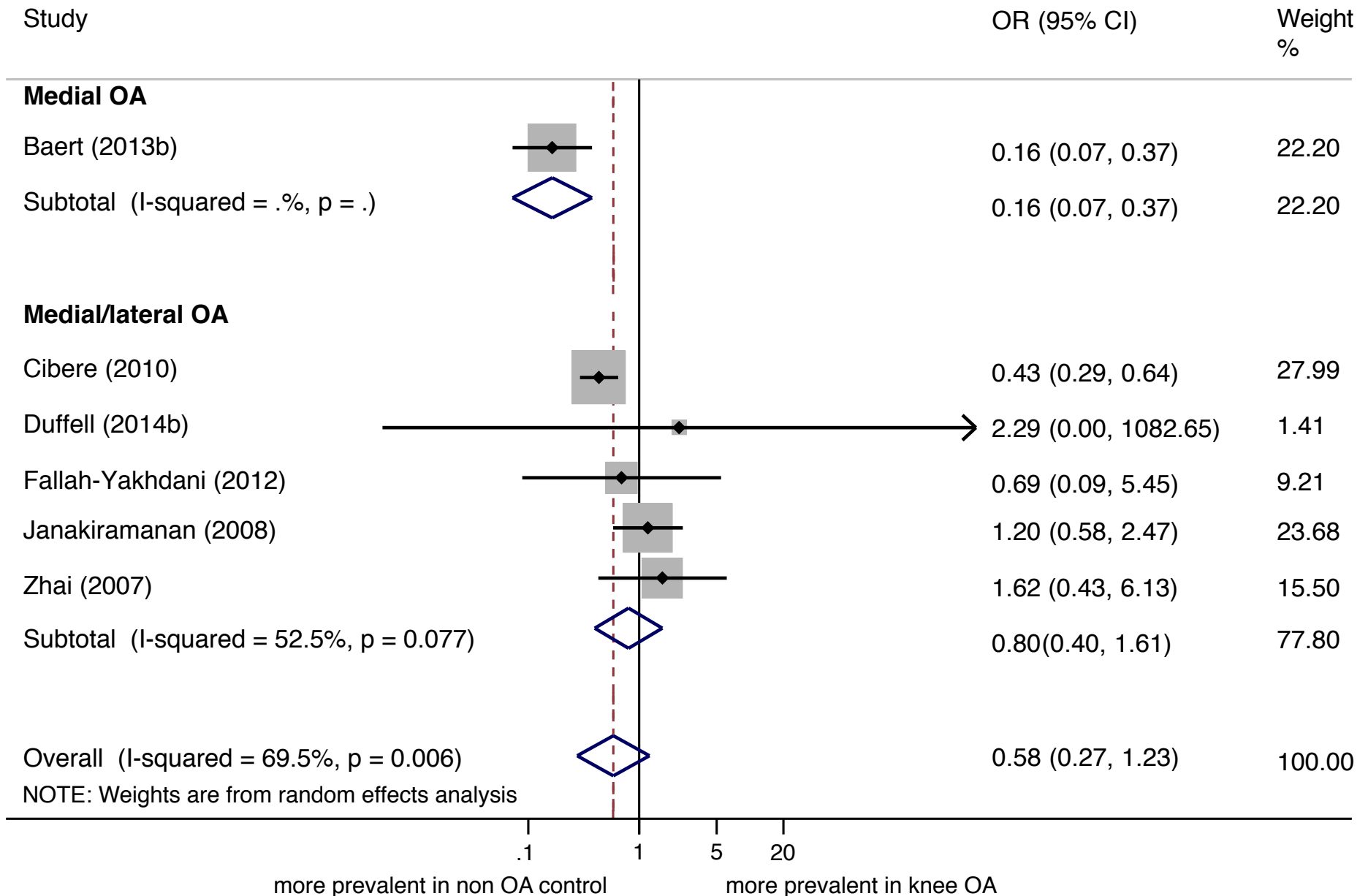

Supplement: Supplementary file 2 — Forest plots of data pooling for skeletal malalignment, muscular dysfunction, impaired proprioception, laxity and abnormal loading, and the presence of knee osteoarthritis (ZIP 1272 kb). [file 12891_2018_2202_MOESM2_ESM.zip › Add. file 2 figure 2 valgus malalignmentR1.pdf]

# Extensor weakness

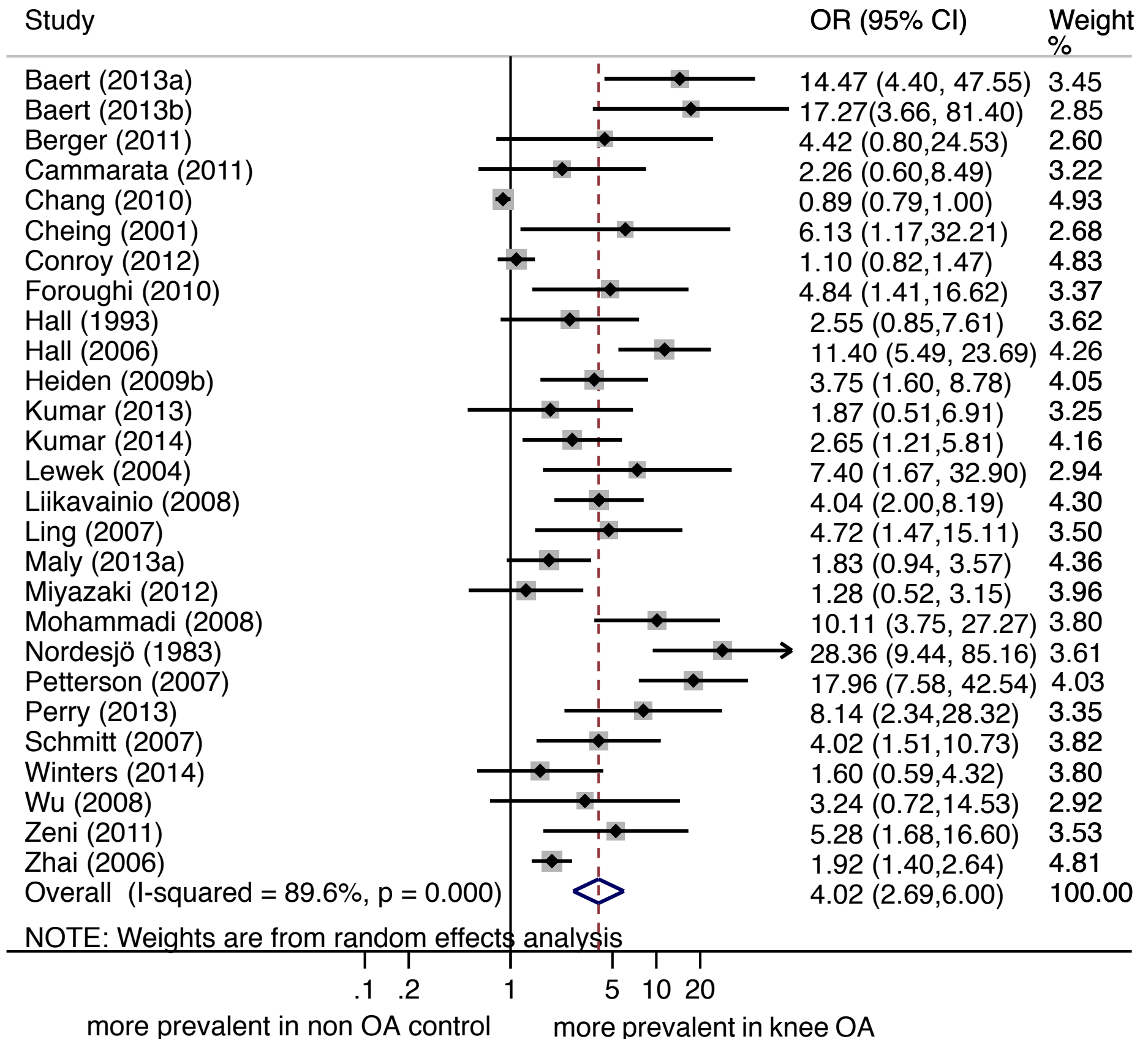

Supplement: Supplementary file 2 — Forest plots of data pooling for skeletal malalignment, muscular dysfunction, impaired proprioception, laxity and abnormal loading, and the presence of knee osteoarthritis (ZIP 1272 kb). [file 12891_2018_2202_MOESM2_ESM.zip › Add. file 2 figure 3 Extensor weaknessR1.pdf]

# Flexor weakness

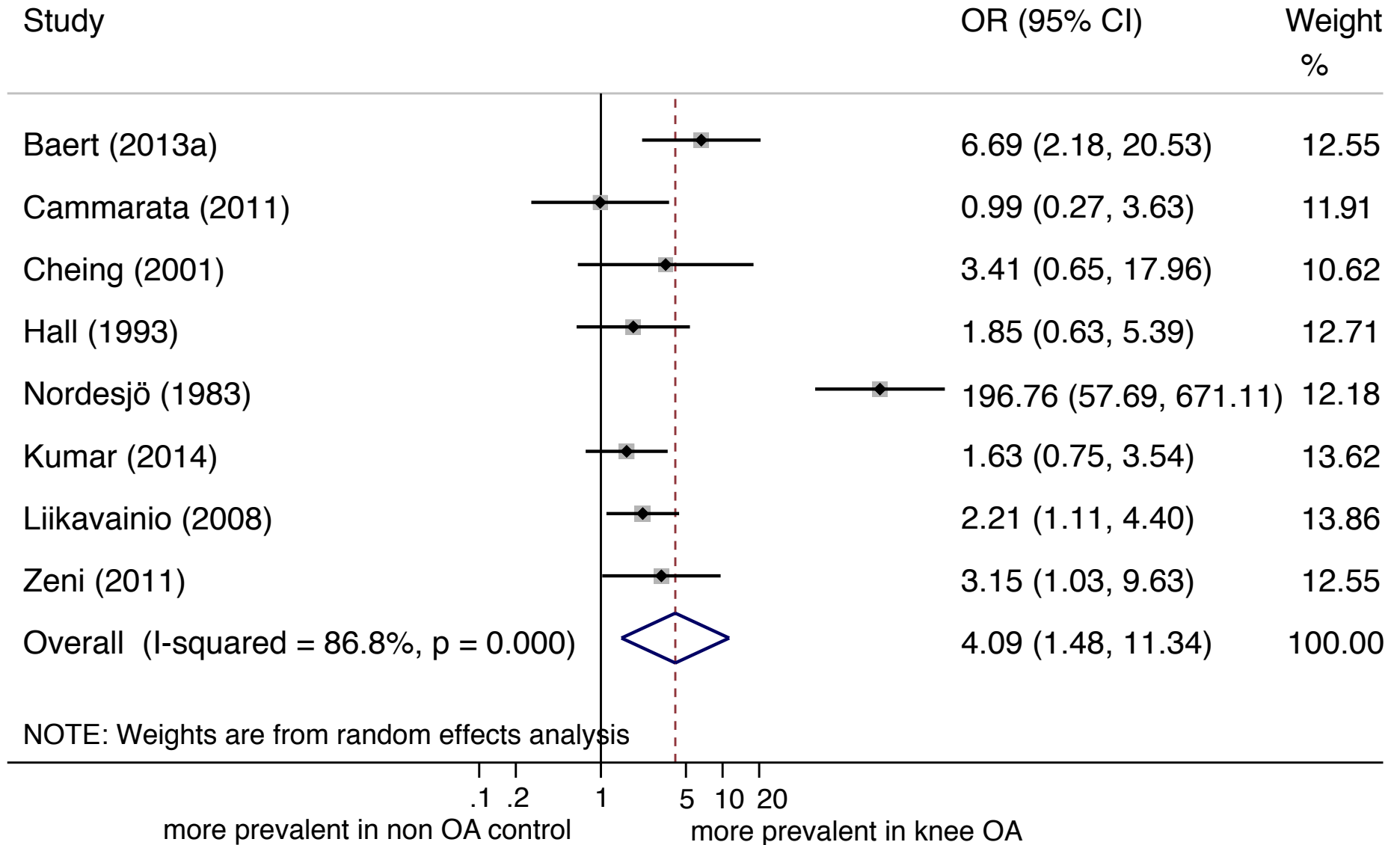

Supplement: Supplementary file 2 — Forest plots of data pooling for skeletal malalignment, muscular dysfunction, impaired proprioception, laxity and abnormal loading, and the presence of knee osteoarthritis (ZIP 1272 kb). [file 12891_2018_2202_MOESM2_ESM.zip › Add. file 2 figure 4 flexor weaknessR1.pdf]

# Reposition error

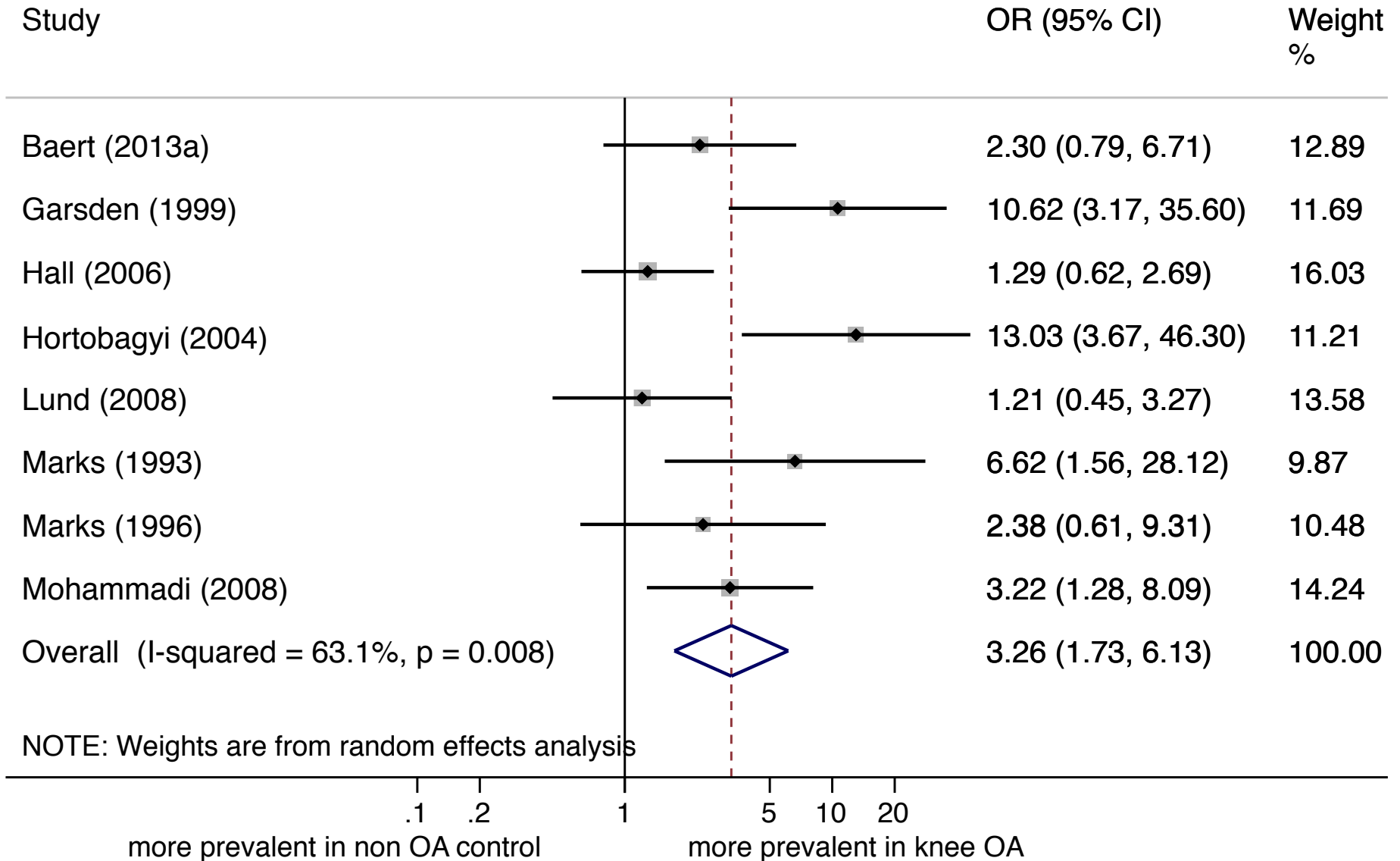

Supplement: Supplementary file 2 — Forest plots of data pooling for skeletal malalignment, muscular dysfunction, impaired proprioception, laxity and abnormal loading, and the presence of knee osteoarthritis (ZIP 1272 kb). [file 12891_2018_2202_MOESM2_ESM.zip › Add. file 2 figure 5 reposition errorR1.pdf]

# Threshold to detect passive movement sagittal plane

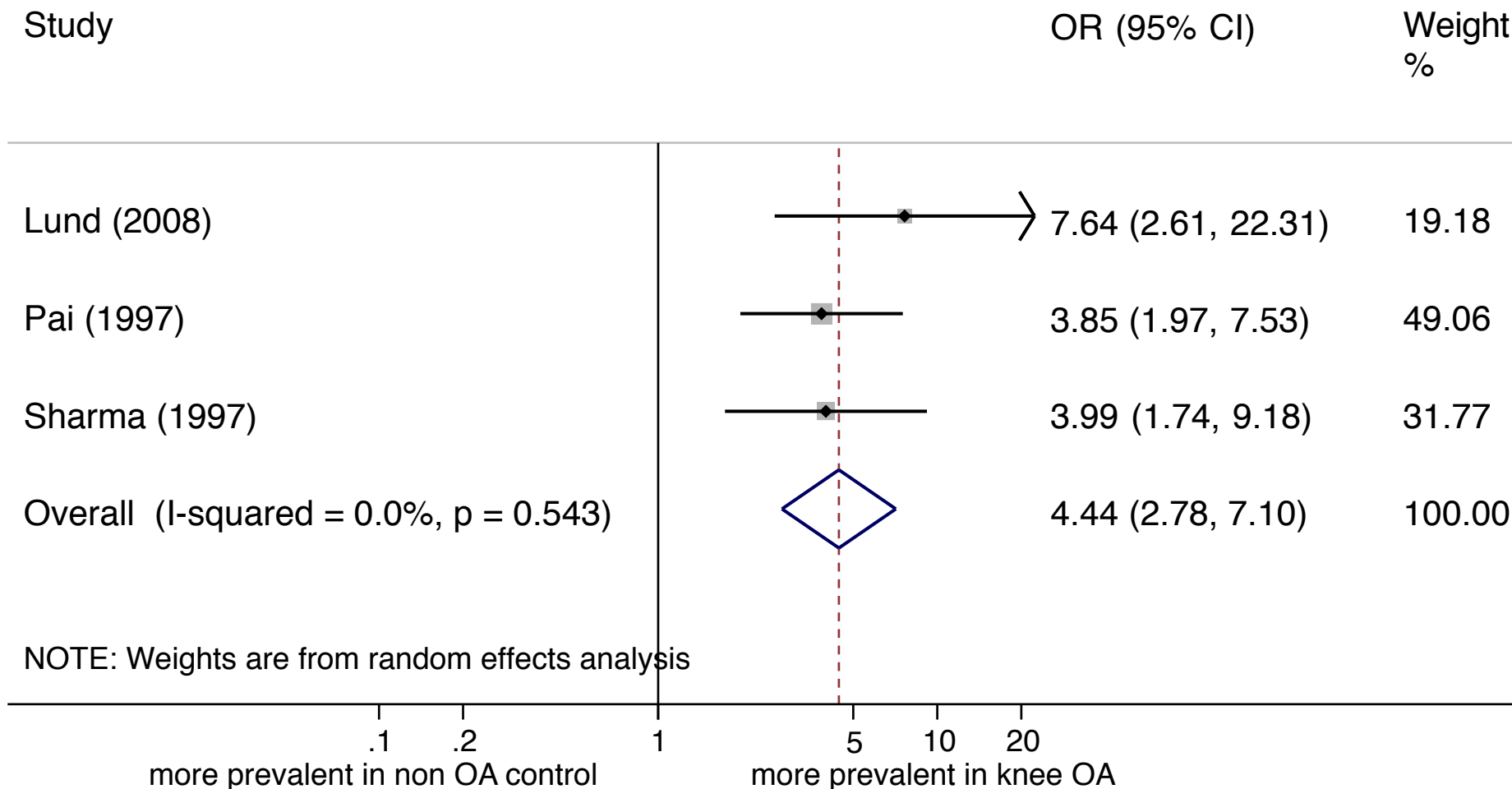

Supplement: Supplementary file 2 — Forest plots of data pooling for skeletal malalignment, muscular dysfunction, impaired proprioception, laxity and abnormal loading, and the presence of knee osteoarthritis (ZIP 1272 kb). [file 12891_2018_2202_MOESM2_ESM.zip › Add. file 2 figure 6 TDPM - sagital planeR1.pdf]

# Threshold to detect passive movement frontal plane - varus direction

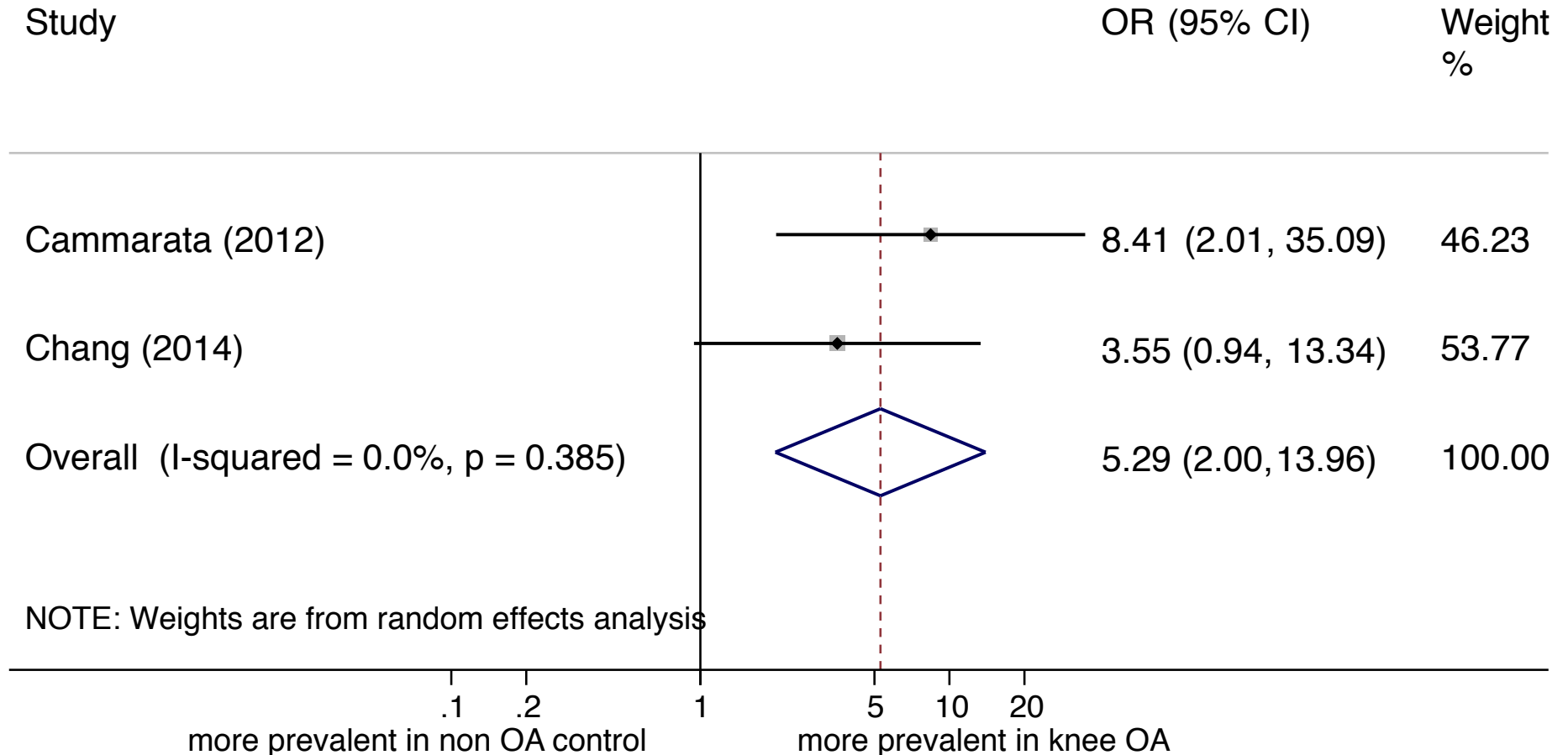

Supplement: Supplementary file 2 — Forest plots of data pooling for skeletal malalignment, muscular dysfunction, impaired proprioception, laxity and abnormal loading, and the presence of knee osteoarthritis (ZIP 1272 kb). [file 12891_2018_2202_MOESM2_ESM.zip › Add. file 2 figure 7 TDPM - frontal plane - varusR1.pdf]

# Threshold to detect passive movement frontal plane - valgus direction

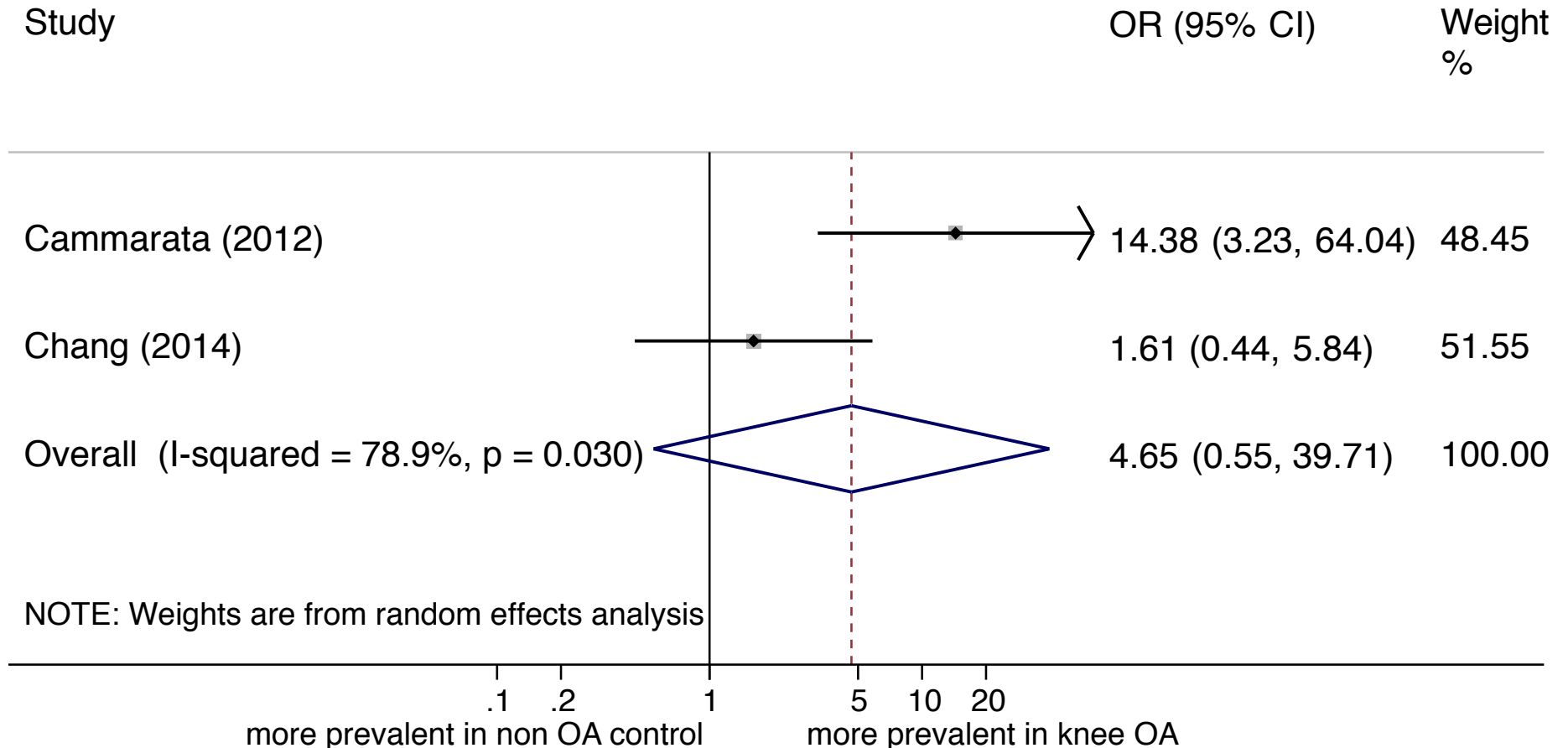

Supplement: Supplementary file 2 — Forest plots of data pooling for skeletal malalignment, muscular dysfunction, impaired proprioception, laxity and abnormal loading, and the presence of knee osteoarthritis (ZIP 1272 kb). [file 12891_2018_2202_MOESM2_ESM.zip › Add. file 2 figure 8 TDPM - frontal plane - valgusR1.pdf]

# Varus-valgus laxity measured at medial side

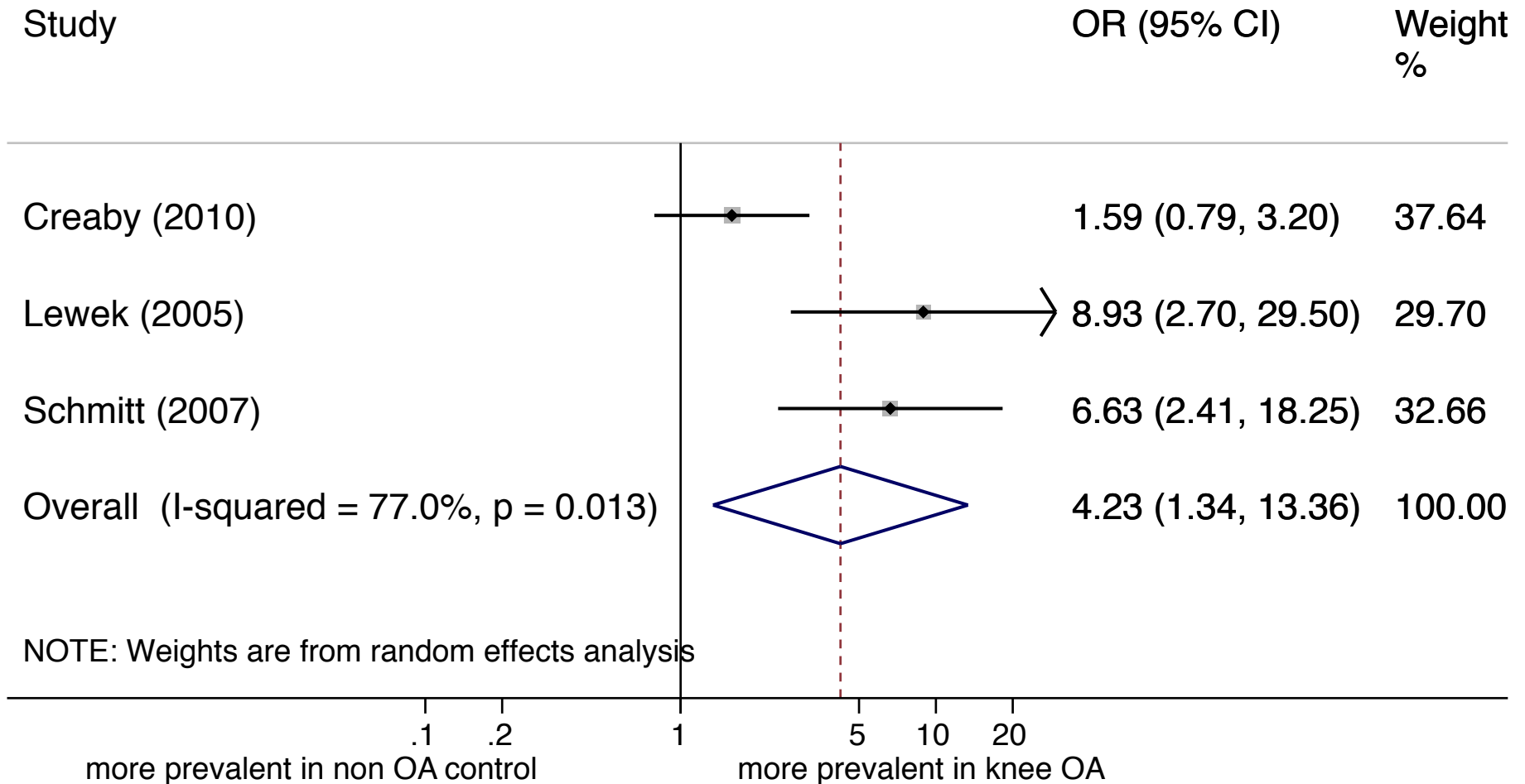

Supplement: Supplementary file 2 — Forest plots of data pooling for skeletal malalignment, muscular dysfunction, impaired proprioception, laxity and abnormal loading, and the presence of knee osteoarthritis (ZIP 1272 kb). [file 12891_2018_2202_MOESM2_ESM.zip › Add. file 2 figure 9 varus-valgus laxity medialR1.pdf]
