# Supplementary material for: Association of malalignment, muscular dysfunction, proprioception, laxity and abnormal joint loading with tibiofemoral knee osteoarthritis - a systematic review and meta-analysis
Source: BMC Musculoskelet Disord. 2018 Jul 28;19:273. doi: 10.1186/s12891-018-2202-8 (PMC6064629; doi:10.1186/s12891-018-2202-8)
Supplement: Supplementary file 3 — Risk of bias and individual study quality for the included studies (DOCX 73 kb). [file 12891_2018_2202_MOESM3_ESM.docx]

**ADDITIONAL FILE 3.**

The risk of bias for the six areas 1) participation, 2) attrition, 3) prognostic factor measurement, 4) outcome measurement, 5) confounding and 6) statistical analysis and reporting was rated as low, moderate or high. * Attrition was not applicable (n/a) for cross-sectional studies. Studies were classified as having low risk of bias if the risk of bias was low or moderate in all areas. Studies with a high risk of bias for at least one area were classified as studies with a high risk of bias.

| Study | Participation | Attrition* | Prognostic factor measurement | Outcome measurement | Confounding | Statistical analysis and reporting | Risk of bias |
| --- | --- | --- | --- | --- | --- | --- | --- |
| Nordesjö (1983) | high | n/a | high | moderate | high | high | high |
| Hall (1993) | high | n/a | moderate | high | low | low | high |
| Marks (1993) | high | n/a | moderate | moderate | moderate | low | high |
| Marks (1996) | high | n/a | moderate | moderate | high | low | high |
| Cooke (1997) | moderate | n/a | low | high | high | low | high |
| Pai (1997) | low | n/a | moderate | low | moderate | low | low |
| Sharma (1997) | low | n/a | moderate | low | moderate | low | low |
| Garsden (1999) | high | n/a | low | moderate | moderate | moderate | high |
| Cheing (2001) | low | n/a | low | low | low | low | low |
| Kaufman (2001) | low | n/a | low | moderate | moderate | low | low |
| Hortobagyi (2004) | low | n/a | low | low | low | low | low |
| Lewek (2004) | moderate | n/a | low | high | low | low | high |
| Lewek (2005) | high | n/a | low | moderate | moderate | moderate | high |
| Aoda (2006) | high | n/a | high | moderate | high | moderate | high |
| Hall (2006) | low | n/a | low | low | moderate | low | low |
| Zhai (2006) | low | n/a | high | high | high | low | high |
| Ling (2007) | moderate | n/a | low | moderate | low | low | low |
| Petterson (2007) | low | n/a | low | high | low | low | high |
| Rudolph (2007) | high | n/a | low | moderate | low | moderate | high |
| Schmitt (2007) | low | n/a | moderate | low | low | low | low |
| Thorp (2007) | low | n/a | low | low | low | low | low |
| Zhai (2007) | low | n/a | low | high | moderate | high | high |
| Astephen (2008) | moderate | n/a | low | high | moderate | moderate | high |
| Janakiramanan (2008) | low | n/a | low | low | low | low | low |
| Liikavainio (2008) | moderate | n/a | low | low | low | moderate | low |
| Lund (2008) | moderate | n/a | low | low | low | moderate | low |
| Mohammadi (2008) | moderate | n/a | low | low | low | low | low |
| Wu (2008) | high | n/a | low | low | low | low | high |
| Heiden (2009a) | low | n/a | low | low | moderate | low | low |
| Heiden (2009b) | low | n/a | low | moderate | low | low | low |
| Chang (2010) | low | n/a | low | low | low | low | low |
| Cibere (2010) | high | n/a | high | low | low | low | high |
| Dixon (2010) | low | n/a | moderate | moderate | low | low | high |
| Foroughi (2010) | moderate | n/a | moderate | low | low | low | low |
| Linley (2010) | low | n/a | low | low | low | low | low |
| Creaby (2010) | moderate | n/a | low | low | low | low | low |
| Berger (2011) | moderate | n/a | moderate | high | moderate | low | high |
| Butler (2011) | low | n/a | low | low | moderate | low | low |
| Cammarata (2011) | moderate | n/a | low | low | low | low | low |
| Zeni (2011) | moderate | n/a | high | low | low | low | high |
| Cammarata (2012) | moderate | n/a | low | low | low | low | low |
| Conroy (2012) | high | n/a | low | low | low | low | high |
| Fallah-Yakhdani (2012) | high | n/a | low | high | low | moderate | high |
| Levinger (2012) | moderate | n/a | low | moderate | low | low | low |
| Miyazaki (2012) | moderate | n/a | low | low | low | low | low |
| Baert (2013a) | low | n/a | low | low | moderate | low | low |
| Baert (2013b) | moderate | n/a | low | low | low | low | low |
| Kumar (2013) | moderate | n/a | low | low | low | low | low |
| Maly (2013a) | moderate | n/a | low | low | low | low | low |
| Maly (2013b) | high | n/a | low | low | high | low | high |
| Metcalfe (2013) | moderate | n/a | low | high | high | low | high |
| Perry (2013) | high | n/a | low | moderate | low | low | high |
| Sagawa (2013) | low | n/a | low | moderate | low | low | low |
| Chang (2014) | moderate | n/a | low | low | low | low | low |
| Duffell (2014a) | high | n/a | low | high | low | low | high |
| Duffell (2014b) | moderate | n/a | low | high | low | low | high |
| Favre (2014) | low | n/a | low | mod | mod | low | low |
| Kumar (2014) | moderate | n/a | low | low | low | low | low |
| Winters (2014) | moderate | n/a | low | high | moderate | low | high |
